# B chromosomes of multiple species have intense evolutionary dynamics and accumulated genes related to important biological processes

**DOI:** 10.1186/s12864-020-07072-1

**Published:** 2020-09-23

**Authors:** Syed F. Ahmad, Maryam Jehangir, Adauto L. Cardoso, Ivan R. Wolf, Vladimir P. Margarido, Diogo C. Cabral-de-Mello, Rachel O’Neill, Guilherme T. Valente, Cesar Martins

**Affiliations:** 1 Department of Structural and Functional Biology, Institute of Bioscience at Botucatu, Sao Paulo State University (UNESP), Botucatu, SP 18618-689 Brazil; 2 Western Paraná State University (UNIOESTE), Center for Biology Science and Health, Cascavel, PR Brazil; 3 Department of General and Applied Biology, Institute of Biosciences, Sao Paulo State University (UNESP), Rio Claro, SP Brazil; 4 Department of Molecular and Cell Biology, University of Connecticut (UCONN), Storrs, CT USA; 5 Institute for Systems Genomics, University of Connecticut (UCONN), Storrs, CT USA; 6 Bioprocess and Biotechnology Department, Agronomical Science Faculty, Sao Paulo State University – UNESP, Botucatu, SP Brazil

**Keywords:** Supernumerary chromosome, Extra chromosome, Genome, Evolution, Next generation sequencing

## Abstract

**Background:**

One of the biggest challenges in chromosome biology is to understand the occurrence and complex genetics of the extra, non-essential karyotype elements, commonly known as supernumerary or B chromosomes (Bs). The non-Mendelian inheritance and non-pairing abilities of B chromosomes make them an interesting model for genomics studies, thus bringing to bear different questions about their genetic composition, evolutionary survival, maintenance and functional role inside the cell. This study uncovers these phenomena in multiple species that we considered as representative organisms of both vertebrate and invertebrate models for B chromosome analysis.

**Results:**

We sequenced the genomes of three animal species including two fishes *Astyanax mexicanus* and *Astyanax correntinus*, and a grasshopper *Abracris flavolineata,* each with and without Bs, and identified their B-localized genes and repeat contents. We detected unique sequences occurring exclusively on Bs and discovered various evolutionary patterns of genomic rearrangements associated to Bs. In situ hybridization and quantitative polymerase chain reactions further validated our genomic approach confirming detection of sequences on Bs. The functional annotation of B sequences showed that the B chromosome comprises regions of gene fragments, novel genes, and intact genes, which encode a diverse set of functions related to important biological processes such as metabolism, morphogenesis, reproduction, transposition, recombination, cell cycle and chromosomes functions which might be important for their evolutionary success.

**Conclusions:**

This study reveals the genomic structure, composition and function of Bs, which provide new insights for theories of B chromosome evolution. The selfish behavior of Bs seems to be favored by gained genes/sequences.

## Background

B chromosomes (Bs) are additional and non-essential extra chromosomes, which show non-Mendelian inheritance and lack the ability of meiotic pairing unlike the normal A chromosomes [1, 2]. The genomic characterization of Bs has remained elusive, since the discovery of Bs [3] about 112 years ago. It is supposed that around 15% of eukaryotic species contain Bs [4], including animals, plants and fungi reported thus far [5], but several questions about their evolutionary origin and function remain unanswered. At first, Bs were presumed neutral or genetically inert elements of the genome, but later it was found that Bs may have either a detrimental or beneficial role (reviewed in Ahmad and Martins [2]). Bs have been found to decrease fertility in maize [6, 7], whereas in extreme situations they may also reduce the genome fitness and eliminate all paternal chromosomes in the wasp *Nasonia vitripennis* [8], and can contain protein coding genes [9, 10]. Although the classical view of Bs as a selfish element has been evoked in many cases, there are relatively few studies that have reported any detrimental effect associated with Bs. Recent studies have provided the new perspective that Bs are not genetically silent, but rather, can carry transcriptionally active copies of rDNA sequences [11] and protein-coding gene [12].

B chromosomes have been reported in 744 animal species including insects, mammals and fishes (http://www.bchrom.csic.es [5]). In addition to other vertebrates with Bs, *Astyanax* fish have emerged as an exciting model for B chromosome research. This genus has been extensively investigated for chromosomal analysis because of the high prevalence of polymorphisms including diverse B chromosomes morphotypes, which have been found in 14 species [13–16]. One of these species, *A. correntinus* contains 36 chromosomes [17] and includes the presence of a macro B chromosome (unpublished data). Another *Astyanax* species with Bs is *A. mexicanus*, commonly recognized as blind cavefish or Mexican tetra that inhabit cave regions, present troglomorphic traces and is an attractive model of evolutionary biology and development studies [18, 19]. The cytogenetics characterization of the blind cavefish revealed a karyotype comprising 2n = 50 chromosomes with 1 or 2 microBs [20]. Although Bs have been extensively investigated in *Astyanax*, including genomic studies, the analysis were mostly focused on cytological observations and also in the investigation of repeated DNAs [21]. However, no study has presented deeper genomic view of the B gene content and therefore analysis is required to reveal the genomic contents and understand B chromosome biology of this organism.

Among insects, grasshoppers (Orthoptera) are another interesting group, in which B chromosomes were investigated in a huge number of species [22]. The grasshopper *Abracris flavolineata* had been previously investigated using cytogenetics methods and contains 2n = 23, X0 (males) sex chromosomes and one or two B chromosomes [23]. Over the years, information has been accumulating for grasshoppers regarding B chromosome population dynamics and their possible origin. However, the knowledge obtained about the molecular composition of B chromosomes in this group of species, focus on the characterization of B repetitive genomic content [24–26].

The cytogenetic analysis of B specific sequences using fluorescence in situ hybridization (FISH), including most of the repetitive DNA types such as dispersed and tandem repeats, rDNA sequences and histones genes remained a primary interest of most studies during the decades of 1990–2010 [27–33]. Furthermore, the genetic composition of isolated Bs in different species was facilitated by flow-sorting and micro-dissection techniques [34]. However, these techniques provide limited material and thus do not fully reveal the relationship of homologous sequences between A and B chromosomes and the complete gene content of Bs. During the last decade, next generation sequencing (NGS) technologies have substantially elevated B chromosome research into a new era of “B-omics” [2]. The multi-omics revolution has offered new opportunities to resolve the classical limitations of cytogenetics analyses. The pioneer study that applied NGS analysis to the study of B chromosome content concluded that the rye B is enriched in pseudogenes as well as different repeat elements [35]. Similarly, a comprehensive genomic analysis of the B chromosome in the cichlid fish, *Astatotilapia latifasciata* discovery that the B comprises of thousands of fragmented genes as well as potentially transcriptional active intact genes [36]. Later, evidence was found that the Bs of the grasshopper, *Eyprepocnemis plorans,* harbor at least ten genes, among which five genes are expressed [37]. Recently, genomics and transcriptomics based analyses have found several transcriptionally active sequences on the B chromosomes [37–43]. Taken together, these findings have sparked an exciting debate about the genomic composition, function and evolution of Bs. Here, we sequenced and analyzed the B carrier genomes of the insect *A. flavolineata* and the fishes *A. correntinus* and *A. mexicanus* to reveal their B-linked repetitive and gene content, to test the hypothesis that the B chromosome accumulates sequences from its host genome for its selfish transmission, and to investigate if the preferential accumulation of these sequences is a conserved feature in multiple species. We found evidences that considerable amount of genomic portions have been migrated from A chromosomes to B via transpositions, duplications and rearrangements events. Unlike classical theories that B chromosomes are gene poor, we found that they are gene rich and contain many protein-coding genes. It seems that B chromosomes tend to gain sequences that are crucial for their own establishment inside the cell. Besides the genes that may give transmission advantage to Bs, there are others coding for many important biological processes.

## Results

### NGS data and coverage-ratio analysis detect sequences on the B chromosomes

**Fig. 1:**
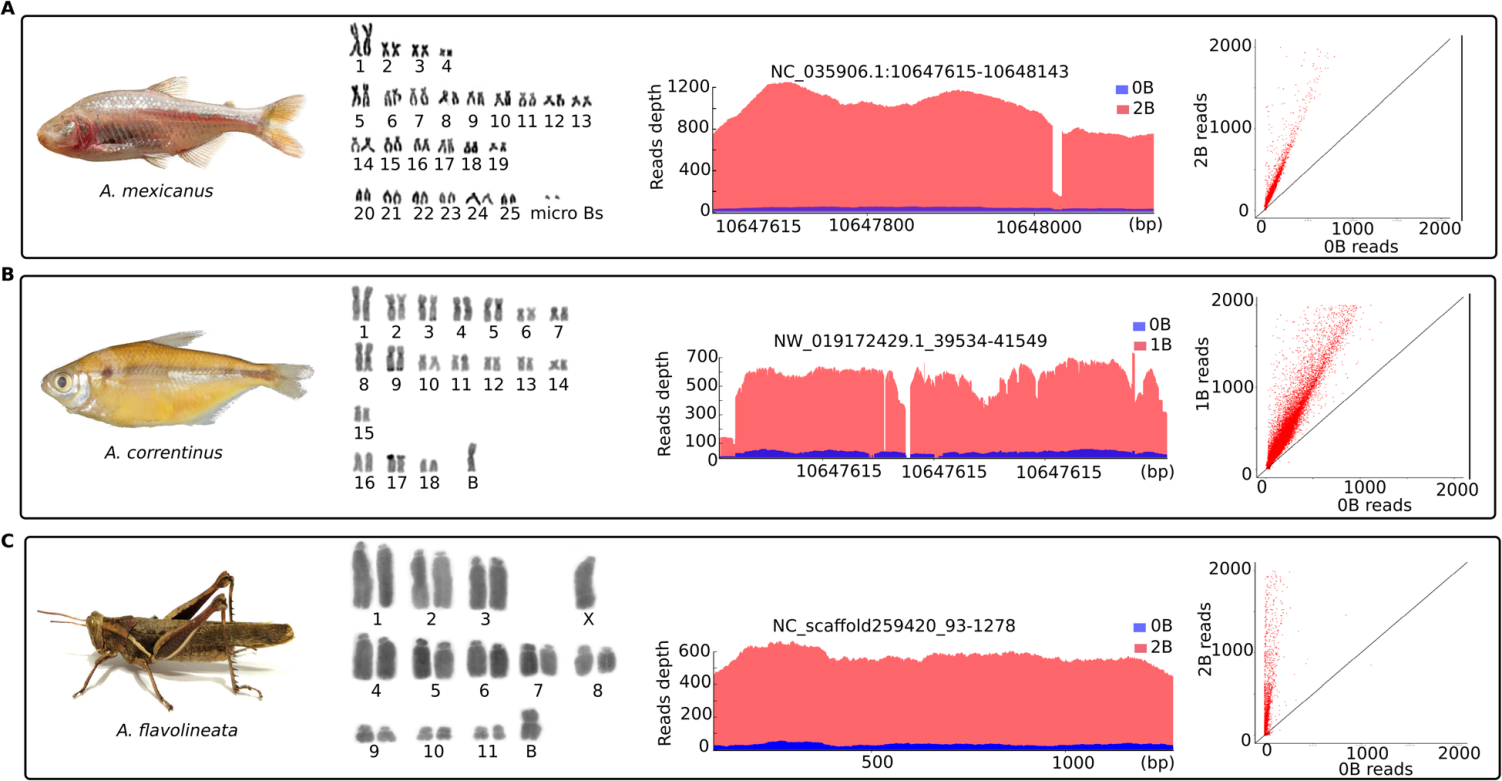
Sequenced species with B chromosomes and their karyotypes and genomic data. **a** *A. mexicanus*, **b** *A. correntinus* and **c** *A. flavolineata*. The plots are given for each corresponding species to show the comparison between B- (0B) and B+ (1B and 2B) coverage. The significant higher coverage of B+ (red peaks) as compared to B- (blue peaks) indicates the amplified genomic region on the B chromosome and extremely underrepresented sequence of this region on the A chromosomes. The X-axis and Y-axis represent read depth and genomic position of the B-block. The blocks are named according to their position in the respective genome assembly. The scatterplots provide the comparison of read abundance for the extracted blocks (upto 2000 reads) between the B- and B+ genomes. Each red dot in these plots is a single block, with X-axis and Y-axis representing the number of mapped reads for B- and B+ genomic libraries. Notice that the blocks above the diagonal lines inclining towards the Y-axis, providing evidence to the extracted B-blocks with higher reads coverage of B+ harbor extra copies of these sequences on B chromosomes

The karyotype analysis identified diploid chromosome numbers (without B) of 36 and 50 for *A. correntinus* and *A. mexicanus*, respectively. The 2n = 36 for *A. correntinus* consists of 12 metacentric, 16 submetacentric, 2 subtelocentric, and 6 acrocentric chromosomes while the 2n = 50 for *A. mexicanus* consists of 8 metacentric, 18 submetacentric, 12 subtelocentric, and 12 acrocentric chromosomes. A large sub-metacentric B chromosome was found in 9 (5males and 4 females) out of 21 samples of *A. correntinus* karyotyped. For *A. mexicanus*, a tiny dot shaped B micro-chromosome was detected in *A. mexicanus* (Fig. 1a, b). Interestingly, across a total of 39 analyzed individuals, the B in *A. mexicanus* is found only in males, whereas no female with B was found, therefore indicating a possible B male-specificity. Out of the 39 karyotyped individuals, 12 were ‘B+ males’, 8 ‘B- males’ and 19 ‘B- females’. In some individuals of *A. mexicanus* we observed 2B micro-chromosomes. The karyotype analyses of the grasshopper *A. flavolineata* (Fig. 1c) confirmed 1 or 2 submetacentric B chromosomes. A total of 69 individuals including 32 males and 37 females were karyotyped, out of which 14 samples had 1B while only 3 samples were found to carry 2B. The male regular karyotype is comprised of 2n = 23 without a B chromosome (14 subtelocentric + X chromosome subtelocentric + 4 submetacentric + 4 metacentric).

We sequenced a total of 8 samples across all 3 species which generating data of around 124×, 82× and 28.1× total sum of all individuals coverage for *A. mexicanus*, *A. correntinus* and *A. flavolineata* respectively (Table 1). The mapping of filtered reads to reference genomes (both from database and de novo assembled) resulted an overall alignment rate of around 92, 91 and 95% for *A. mexicanus*, *A. correntinus* and *A. flavolineata.* The B chromosome sequencing reads were mapped to reference genomes; hence the mapped reads representing a B chromosomal region in B+ sample would have an increase coverage level as compared to the aligned reads in B- sample (see summary of coverage detection steps as Supplementary Fig. S1). These regions having remarkably higher coverage, called B blocks, were detected in the genomes of each of the three model species (Fig. 1; Supplementary Figs. S2 and S3). We also performed *de novo* assembly of the genomes for *A. mexicanus, A. correntinus* and *A. flavolineata* (Supplementary Table S1).

**Table 1:** NGS data generated for the analysis of B chromosomes in the present study

Genome IDs	Sample	Number of raw reads	Reads length	Coverage	Number of filtered reads	Coverage after filtration	Genome size
ame-1475-0b	*A. mexicanus* (0B)	265,761,096	151 bp	40.1×	265,374,050	40.07×	1.5 Gb
ame-1465-1b	*A. mexicanus* (1B)	265,789,908	151 bp	40.13×	265,402,766	40.07×	
ame-1466-2b	*A. mexicanus* (2B)	358,144,230	151 bp	54.07×	357,697,838	54.01×	
aco-2220-0b	*A. correntinus* (0B)	388,448,208	151 bp	31.46×	387, 817,038	31.1×	2.3 Gb
aco-2749-1b	*A. correntinus* (1B)	506,354,302	151 bp	41.01×	505,608,854	40.95×	
afl-1 h-2 h-4 h-0b	*A. flavolineata* (0B)	836,480,126	151 bp	19.95×	834,822,322	19.91×	6.3 Gb
afl-5 h-6 h-1b	*A. flavolineata* (1B)	453,566,904	151 bp	10.81×	452,662,368	10.79×	
afl-7 h-8 h-2b	*A. flavolineata* (2B)	431,189,476	151 bp	10.28×	430,174,870	10.26×	

### Genomic characterization of the B chromosome uncovers its gene contents with diverse functions

**Fig. 2:**
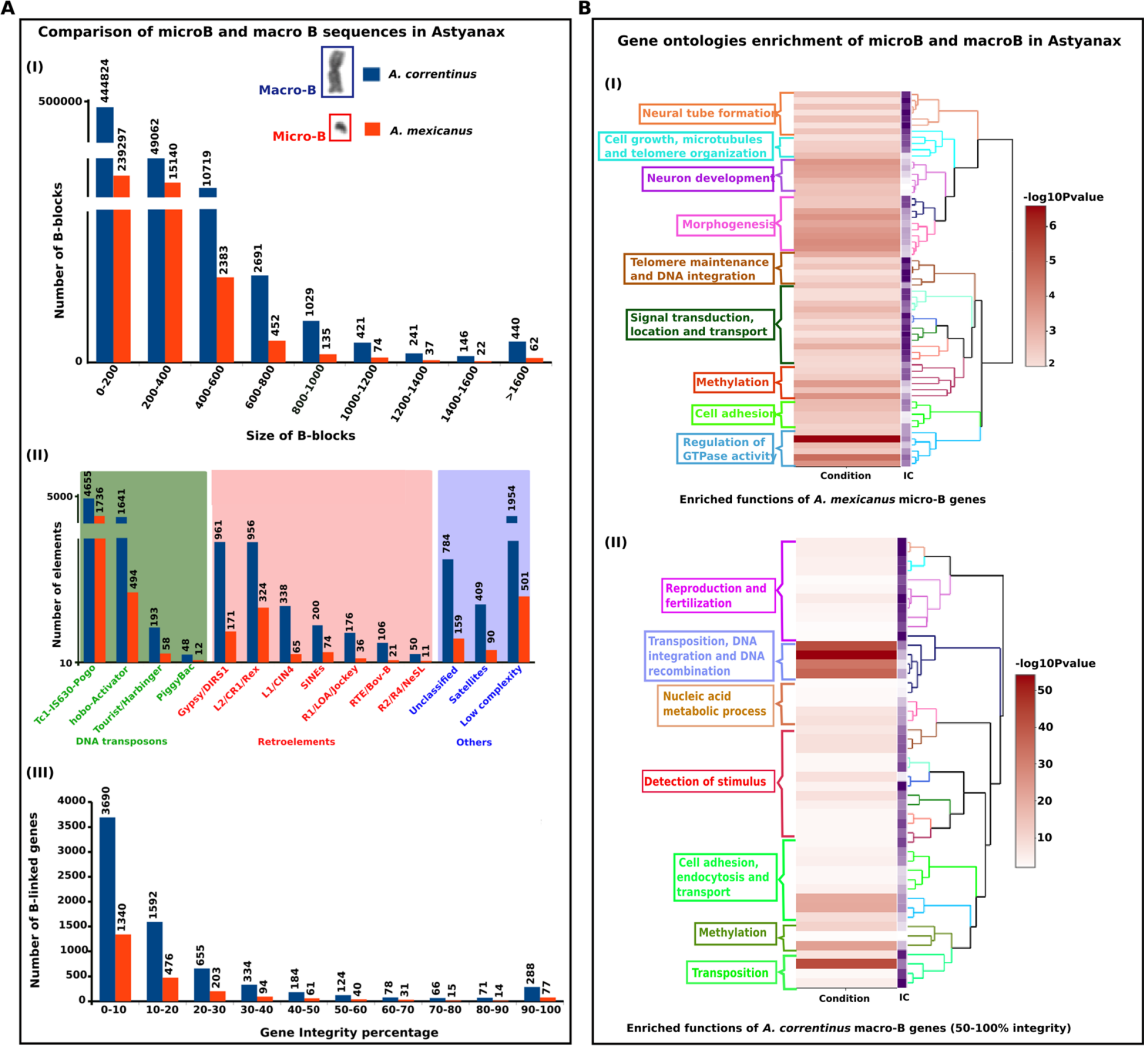
Characterization of genomic regions detected on the microB of *A. mexicanus* and macroB of *A. correntinus*. **a** (I) A comparison between B blocks number with respective blocks size range. (II) Repeat annotations of B blocks showing the abundance of diverse classes of DNA transposons on the B chromosomes. (III) Results of gene integrity analysis showing the number of genes (Y axis) in each integrity percentage group (X axis). The bar charts are scaled with breaks to normalize difference in data. **b** Heatmap plots and clustering visualization of GO enrichment of gene contents on microB (I) of *A. mexicanus* and macroB (II) of *A. correntinus*. The enriched functions are colored according to the dendogram of GO clusters based on the semantic similarity distance and ward.D2 criteria. The higher the -log10 *P*-value, the more enriched the functions. Notice the enrichment of functions associated with DNA integration, DNA recombination, transposition, cell growth, microtubules organization and telomere maintenance that might have provided transmission advantage and formation of a protoB

A total of 509,028 and 257,784 and 9845 number of B-blocks were recorded for *A. correntinus*, *A. mexicanus* and *A. flavolineata*, respectively. A list of the B-blocks with sequence length of at-least 200 bp or more is listed as Supplementary dataset (Supplementary dataset 1). For gene annotations of *A. correntinus* and *A. mexicanus*, blocks under 200 bp were removed, leaving a total of 64,627 and 18,340, respectively. B-blocks ranged in length from 200 bp to 10 kb (Fig. 2a). There were multiple regions in the B+ genomes with multiple B blocks in close proximity to one another, suggesting that a larger region was likely transferred to the B chromosome as a whole segment rather than as multiple smaller segments. Sequencing of B+ samples yielded a lower genome coverage (around 10×) for *A. flavolineata* due to its large estimated genome size (6.3 Gb; Table S1), thus we could not derive a comprehensive list of B-blocks, and subsequent gene integrity analysis, for this species. However, while incomplete, we were able to find a considerable amount of B chromosome sequence for this species (Supplementary Fig. S4).

The repeat characterization using RepeatExplorer [44] pipeline concluded a total of 65 and 35% comprising of repeats in *A. correntinus* and *A. mexicanus* in the full genomes respectively. A search for repetitive DNAs in the B chromosome blocks found different types of repeats distributed across various blocks. In the blocks of *A. correntinus*, approximately 5.78% of repeats are retroelements, 3.38% are LTR elements with a higher amount of Gypsy/DIRS1, and 2.94% are DNA transposons dominated by Tc1-IS630-Pogo elements. In *A. mexicanus*, the blocks are comprised of 7.7% retroelements, 3.14% of LTR and 15.67% of DNA transposons. Despite a lacking a high amount of B chromosome sequencing coverage, we detected an abundance of R1/LOA/Jockey, Tc1-IS630-Pogo and Gypsy/DIRS1 on the B chromosome of *A. flavolineata* (Supplementary Fig. S4).

To identify the intact genes on the B chromosomes, we calculated an integrity score for each gene sequence annotated in the B-blocks. The majority of B-located genes of *A. correntinus* (91%) and *A. mexicanus* (93%) have integrity scores < 50% (Fig. 2a; Supplementary dataset 2). The NGS data analysis indicating the higher number of B-blocks and number of repeats and genes for *A. correntinus* as compared to *A. mexicanus* (Fig. 2a) coincides with the karyotype data with respect to B chromosome size.

The functional annotation of genes detected on the B chromosomes was determined and gene ontologies enrichment was performed. We considered the complete list of genes (including both fragmented and integral) for the microB of *A. mexicanus*. Only genes with an integrity percentage > 50% were considered for the macroB of *A. correntinus* due to the large number of gene fragments observed. The GO analysis for these genes on both microB and macroB revealed a enrichment for genes in cellular processes, such as microtubule processes, transpositions, recombination, and telomere maintenance, all groups with remarkably high -log10 *P*-values (Fig. 2b). These functions are significantly over represented on both microB and macroB, which indicate that the B chromosome tends to gain gene contents to maintain its transmission in cell division and facilitate its evolutionary success.

We retrieved a list of high integral genes detected on the Bs that are directly involved in chromosome formation and cell cycle related functions (Table 2). These cell cycle genes were found in all of our analyzed species indicating their importance in the establishment and maintenance of Bs.

**Table 2:** A list of genes detected on the B chromosomes of A. correntinus, A. mexicanus and A. flavolineata involved in chromosomes, microtubules or cell cycle related processes

Species	Ensembl ID	Protein name	Gene name	Gene description	Function related to cell cycle/chromosome
***A. correntinus***	ENSAMXT00000047800.1	ATP-dependent DNA helicase Q1	RECQL	DNA helicase activity	Chromosome organization
	ENSAMXT00000008370.2	Structural maintenance of chromosomes 2	smc2–201	Structural maintenance of chromosomes 2	Chromosome organization
	ENSAMXT00000050221.1	Histone-lysine N-methyltransferase	ASHH2	Histone lysine methylation	The modification of a histone by addition of one or more methyl groups to a lysine residue.
	ENSAMXT00000055649.1	E3 ubiquitin-protein ligase	RNF8	Ubiquitination of histones H2A and H2AX	Chromatin decondensation
	ENSAMXT00000043499.1	dkey-16p21.7	dkey-16p21.7	Protein kinase activity	Chromatin remodeling
	ENSAMXT00000048305.1	SWI/SNF-related matrix-associated actin-dependent regulator of chromatin subfamily E member 1	SMARCE1	Alteration of DNA-nucleosome topology	Chromosome remodeling
	ENSAMXT00000039111.1	ch211-255f4.7	Novel gene	unknown	Chromatin remodeling
	ENSAMXT00000039111.1	CCCTC-binding factor (Zinc finger protein)	CTCF	Chromatin biding	Regulation of genne expression
	ENSAMXT00000034475.1	Zgc:85722	Zgc:85722	Unkonown	Establishment of mitotic spindle orientation, microtubule-based process, cell cycle
	ENSAMXT00000030346.1	Tubulin alpha chain	TUBA	It binds two moles of GTP, one at an exchangeable site on the beta chain and one at a non-exchangeable site on the alpha chain.	Major constituent of microtubules
	ENSAMXT00000030102.1	microtubule-associated proteins	MAPs	Microtubules cytoskeleton organization	Stability and regulate microtubules,
	ENSAMXT00000026549.2	MAP7 domain-containing protein 1	MAP7D1	Microtubule cytoskeleton organization	Stability and regulate microtubules,
	ENSAMXT00000057964.1	Histone H4	HIST1H4J	Core component of nucleosome. Nucleosomes wrap and compact DNA into chromatin	Chromosome stability
***A. mexicanus***	ENSAMXT00000054831.1	Eukaryotic translation initiation factor 4 gamma 1	EIF4G1	Recognition of the mRNA cap	Mitochondrion organization
	ENSAMXT00000043499.1	dkey-16p21.7	dkey-16p21.7	Protein kinase activity	Chromatin remodeling
***A. flavolineata***	FBtr0070762	DNA replication licensing factor MCM3	Mcm3	Putative replicative helicase	Cell cycle’ and DNA replication initiation and elongation in eukaryotic cells
	FBtr0076253	General transcription and DNA repair factor IIH helicase subunit XPB	hay	ATP-dependent 3′-5′ DNA helicase	Transcription-coupled nucleotide excision repair (NER) of damaged DNA
	FBtr0079901	SWI/SNF-related matrix-associated actin-dependent regulator of chromatin subfamily A containing DEAD/H box 1 homolog	Etl1	ATP-dependent nucleosome-remodeling activity	DNA repair and heterochromatin organization
	FBtr0083193	Meiotic recombination protein	rec	Separation of sister chromatids and homologous chromosomes	Chromosomes separation during meisis
	FBtr0085875	205 kDa microtubule-associated protein	Map205	Phosphorylation	Microtubule assembly and interaction
	FBtr0087461	Structural maintenance of chromosomes protein 3	SMC2	chromosome cohesion during the cell cycle	Cell division
	FBtr0087904	Anastral spindle 1, isoform A	ana1	Nucleation of microtubules	Spindle fiber formation
	FBtr0339505	Meiotic central spindle, isoform B	Meics	Nucleation of microtubules	Spindle Assembly and Chromosome Segregation
	FBtr0304801	Mini spindles, isoform D	msps	Nucleation of microtubules	Integrity of the Mitotic Spindle
	FBtr0299509	Doublecortin-domain-containing echinoderm-microtubule-associated protein, isoform G	DCX-EMAP	Assembly dynamics of microtubules	Microtubules cytoskeleton segregation
	GDIO01044471.1	SWI/SNF-related matrix-associated actin-dependent regulator of chromatin subfamily A-like protein 1	SMARCAL1	ATP-dependent annealing helicase	Catalyzes the rewinding of the stably unwound DNA
	GDIO01001775.1	Histone-lysine N-methyltransferase eggless	egg	Histone methyltransferase	Histones methylation
	GDIO01013035.1	Dosage compensation regulator	mle	Compensation of X chromosome linked genes	Sex differenciation
	GDIO01001774.1	Histone-lysine N-methyltransferase eggless	egg	Histone methyltransferase	Histone H3-K9 methylation, heterochromatin organization, positive regulation of methylation-dependent chromatin silencing
	GDIO01030872.1	GH07148p	Dmel\CG1582	unknown	Chromatin silencing, mitotic chromosome condensation
	GDIO01009316.1	Tubulin beta-1 chain	mec-7	Microtubules formation	Attachment of spindle microtubules to kinetochore involved in meiotic chromosome segregation
	GDIO01018464.1	Gamma-tubulin complex component 3	GCP3	Microtubule nucleation	Spindle assembly, regulation of cell cycle, meiotic nuclear division

**Fig. 3:**
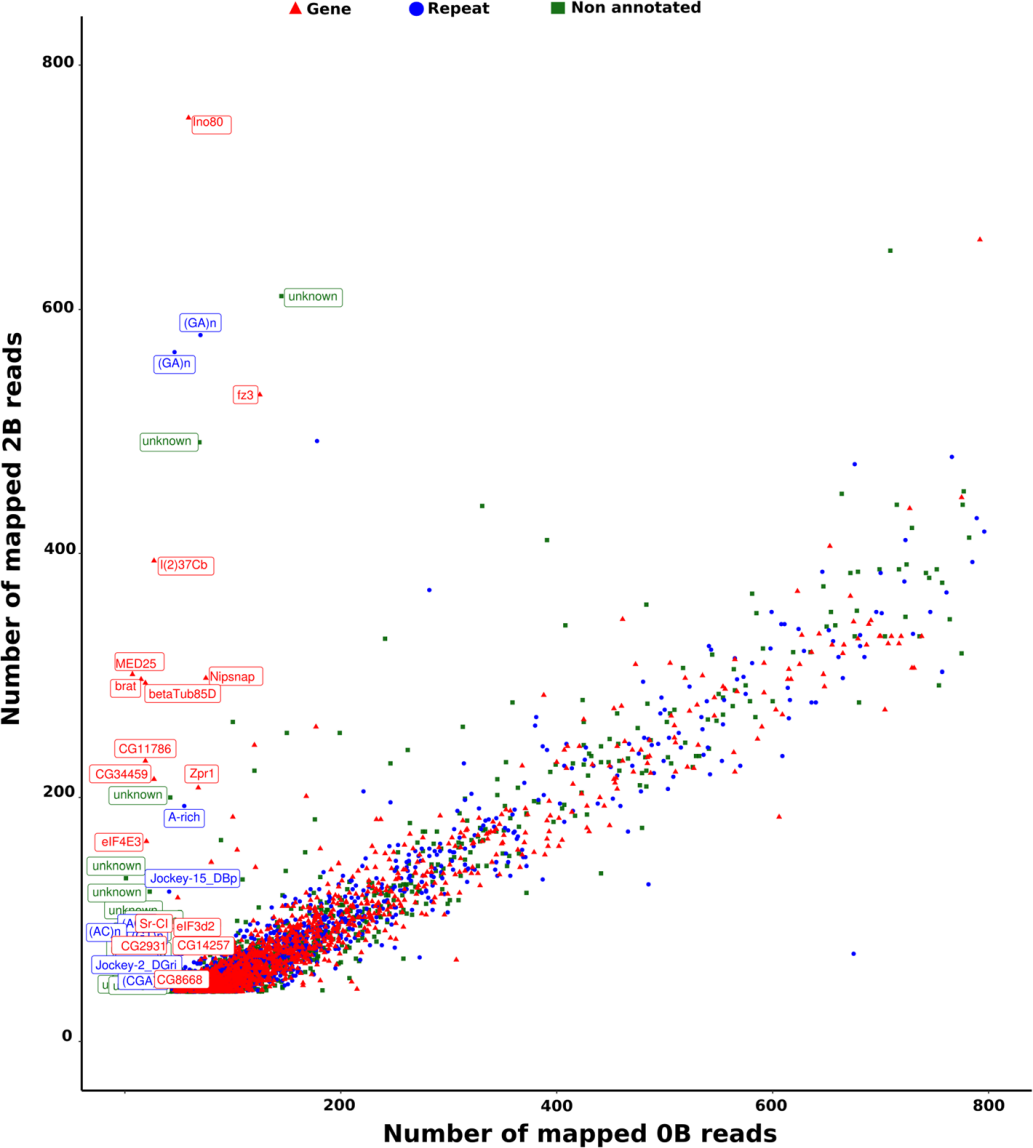
Identification of protein-coding genes located in B chromosomes of *A. flavolineata* using the number of mapped reads that map to the CDSs found in the transcriptome of the 0B (X axis) and 2B (Y axis). Each dot represents a coding sequence. The B-linked CDS (having log2 greater than 1.5) are highlighted with annotation labels. The plot is limited upto 800 mapped reads. The scatter plots showing B linked CDS for Astyanax are given as Supplementary Fig. S5 and S6

To further extend our insights into the protein coding sequences found on the B chromosomes, we employed a comparison based strategy using a difference in the counts of mapped reads against the reference transcript contigs. The mapping of the Illumina reads from the B- and B+ genomes on the coding sequences (CDS) of transcriptomes revealed a total of 38,071 reads for *A. mexicanus*, 34,301 for *A. correntinus* and 3916 transcripts (contigs) for *A. flavolineata* with more than 40 reads mapped for both B- and B+ genomes. Graphical representation of the B- and B+ showed the presence of CDSs over-represented in the B+ genomes (Fig. 3, Supplementary Figs. S5 and S6). Remarkably, a total 100 and 53 CDSs showed a log_2_ 2B/0B quotient > 1.5 for *A. mexicanus* and *A. flavolineata* respectively, and 436 CDS for *A. correntinus* with a log_2_ 1B/0B > 1 i.e. the expected value if each B chromosome carried at least one copy of the CDS (see methods). Annotation revealed that most of these CDSs were orthologous to different protein-coding genes in the reference set of genes while others were also identified as repeat elements. Some of the CDS did not align to the references, therefore we termed them as non-annotated or unknown. The annotation detected several novel genes on the B chromosomes of *Astyanax* species. The CDS with the highest log_2_ quotient representing a high confidence of B chromosome presence are listed a Table 3 and Supplementary dataset 3 for each species. The coverage pattern for some of these CDS were also visualized to confirm the higher peaks for B+ as compared to B- genomes, hence providing evidence of their expanded copy number on B chromosomes (Supplementary Figs. S7, S8, S9).

**Table 3:** Results of mapping gDNA reads from 0B and 1(2)B of A. mexicanus, A. correntinus and A. flavolineata on transcriptome CDS. The higher Log_2_ ratio indicates the highly confident CDSs on B chromosome. See the complete list as Supplementary dataset file 3

Transcript ID	0B reads	1(2)B reads	1(2)B/0B	Log_**2**_ ratio	Annotation
***A. mexicanus***					
ARA0AAA105YI15EM1.b.am.1	277	1496	5.40072	2.43315	tars
ARA0AAA15YI22EM1.b.am.1	44	569	12.9318	3.69285	novel_gene
ARA0AAA117YG08EM1.b.am.1	51	145	2.84314	1.50749	scn12aa
ARA0AAA12YF15EM1.b.am.1	251	968	3.85657	1.94732	exosc7
ARA0AAA15YI22EM1.b.am.1	44	569	12.9318	3.69285	novel_gene
ARA0AAA6YO21EM1.b.am.1	255	816	3.2	1.67807	novel_gene
ARA0AAA75YN07EM1.b.am.1	40	142	3.55	1.82782	zgc
ARA0ABA106YI03EM1.b.am.1	48	165	3.4375	1.78136	novel_gene
ARA0ABA11YM14EM1.b.am.1	61	224	3.67213	1.87662	zgc
ARA0ABA21YO23EM1.b.am.1	90	271	3.01111	1.5903	novel_gene
ARA0ABA22YA20EM1.b.am.1	57	200	3.50877	1.81097	shisal1b
ARA0ABA3YF16EM1.b.am.1	49	178	3.63265	1.86102	ndufa11
ARA0ABA43YF05EM1.b.am.1	135	431	3.19259	1.67473	pcdh1g31
ARA0ABA65YL16EM1.b.am.1	64	189	2.95312	1.56224	novel_gene
ARA0ABA73YP22EM1.b.am.1	76	224	2.94737	1.55943	novel_gene
ARA0ABA93YE09EM1.b.am.1	48	168	3.5	1.80735	novel_gene
ARA0AFA5YA16EM1.b.am.1	119	357	3	1.58496	Gon4l
***A. correntinus***					
ARA0AAA39YG16EM1.b.am*.1*	*727*	*14,308*	*19.681*	*4.29873*	*novel_gene*
ARA0AAA40YD05EM1.b.am*.1*	*156*	*517*	*3.314*	*1.72857*	*jac3*
ARA0ABA10YE08EM1.b.am*.1*	*586*	*1661*	*2.834*	*1.50284*	*arih1l*
ARA0ABA90YO24EM1.b.am*.1*	*117*	*359*	*3.068*	*1.6173*	*novel_gene*
ARA0AFA5YA16EM1.b.am*.1*	*96*	*301*	*3.135*	*1.64847*	*gon4l*
ARA0AHA14YK02EM1.b.am.1	50	151	3.02	1.59455	unknown
ARA0AGA13YG24EM1.b.am.1	89	472	5.303	2.40681	unknown
ARA0ABA7YH17EM1.b.am.1	44	140	3.182	1.66993	unknown
ARA0ABA25YA20EM1.b.am.1	44	126	2.864	1.51803	unknown
ARA0ABA107YI10EM1.b.am.1	42	119	2.833	1.50233	unknown
ARA0ABA103YB02EM1.b.am.1	101	340	3.366	1.75104	unknown
ARA0AAA70YD16EM1.b.am.1	71	318	4.479	2.16318	unknown
***A. flavolineata***					
*GDIO01012001.1*	*68*	*208*	*3.059*	*1.61306*	*Zpr1*
*GDIO01012001.1*	*68*	*208*	*3.059*	*1.61306*	*Zpr1*
*GDIO01014672.1*	*75*	*298*	*3.973*	*1.99023*	*Nipsnap*
*GDIO01044471.1*	*59*	*757*	*12.831*	*3.68156*	*Ino80*
*GDIO01045346.1*	*125*	*530*	*4.24*	*2.08406*	*fz3*
*GDIO01030872.1*	*69*	*491*	*7.116*	*2.83107*	*unknown*
*GDIO01018848.1*	*42*	*200*	*4.762*	*2.25157*	*unknown*
*GDIO01009316.1*	*145*	*611*	*4.214*	*2.07519*	*unknown*

### The Bs harbor unique and exclusive sequences

**Fig. 4:**
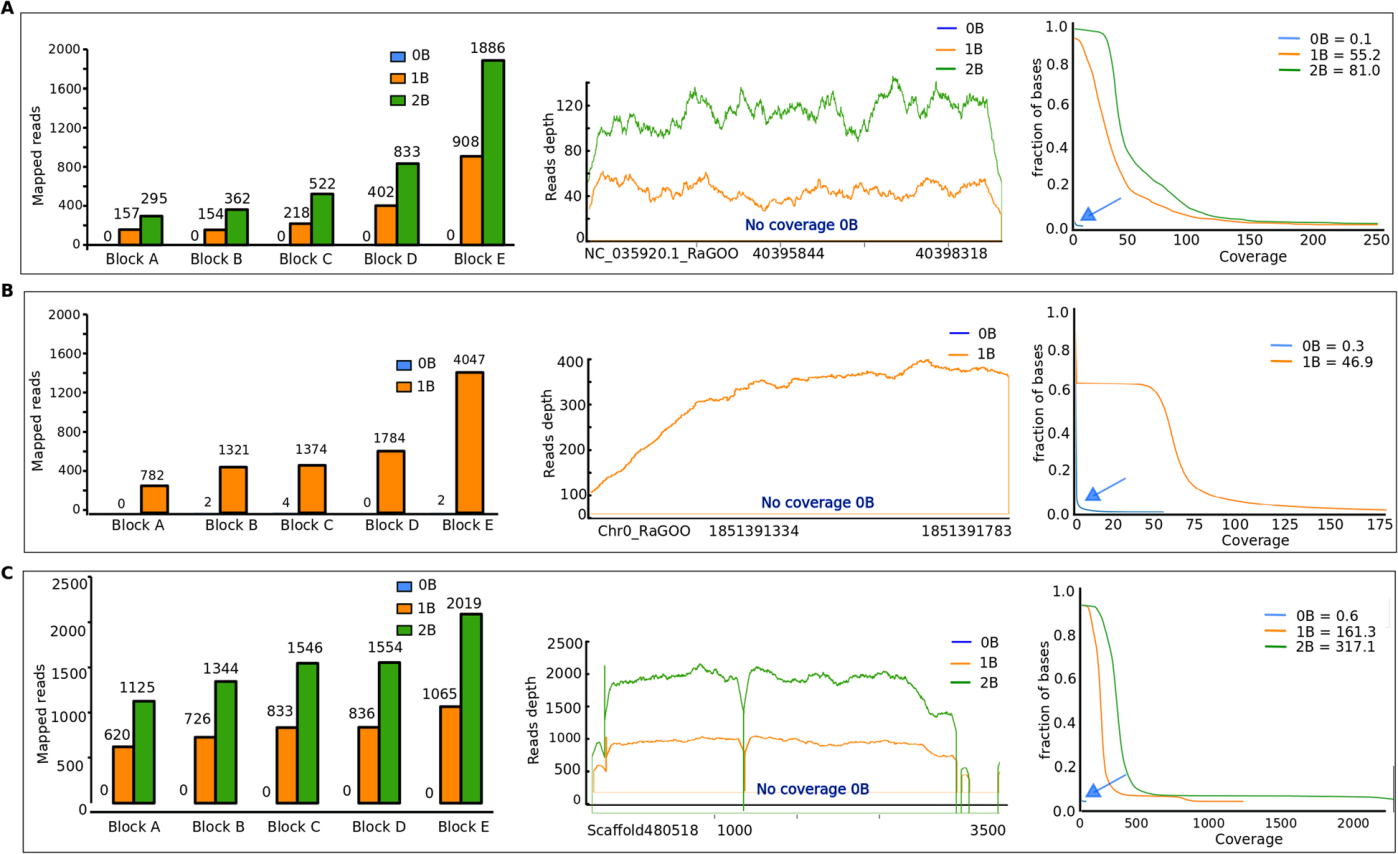
Comparative plots of the exclusive sequences detected on the Bs of (**a**) *A. mexicanus* (**b**) *A. correntinus* and (**c**) *A. flavolineata*. The bar charts (left) of five representative blocks compare the relative abundance of mapped reads for 0B (blue), 1B (orange) and 2B (green) genomes. The coverage plots (middle) as an example block for each species depicts the reads depth confirm the exclusive representation on 1B and 2B genomes. The mean coverage plots (left) of all exclusive blocks show the fraction of the genome with respective coverage. Notice the mapped reads, reads depth and mean coverage of 0B genomes in each species is negligible, thus confirming the absence of these sequences on A chromosomes and specificity to B chromosomes

To reveal the unique sequences found on the B chromosomes, we first performed *de novo* assemblies of the B+ genomes. The reference B+ genome is expected to contain both A and B chromosome sequences; thus the comparison of mapping B- and B+ genomes revealed the B specific sequences. These B-specific sequences were analyzed on the basis of reads mapped to the reference *de novo* assemblies with B chromosomes. The sequences were compared in a way that there were no alignments recorded for B- while the B+ genomic reads should have uninterrupted alignments with minimum 50× coverage in the same region (see methods). From these alignments, a total of 140; 1698; and 247 number of exclusive B regions with a minimum of 200 bp sequence length, were obtained for *A. mexicanus, A. correntinus*, and *A. flavolineata* respectively. We were able to extract sequences up to 3 kb long where alignments of at least 50× coverage were recorded along the complete region (entire sequence covering B) for the B+ but negligible alignments for B- genomes. Interestingly, we found that the number of mapped reads increased proportionally with the number of Bs, (Fig. 4; Supplementary dataset 4) but remain null in B- genomes. Blast search of these exclusive Bs’ sequences against “nr” NCBI database did not return any feasible hit, indicating their unique and novel nature*.* The ‘somewhat similar’ search fit weak hits with mitochondrial genes for most of these sequences in *A. flavolineata*.

### The micro-B of cavefish was invaded by satDNA and amplicon gene like sequences

**Fig. 5:**
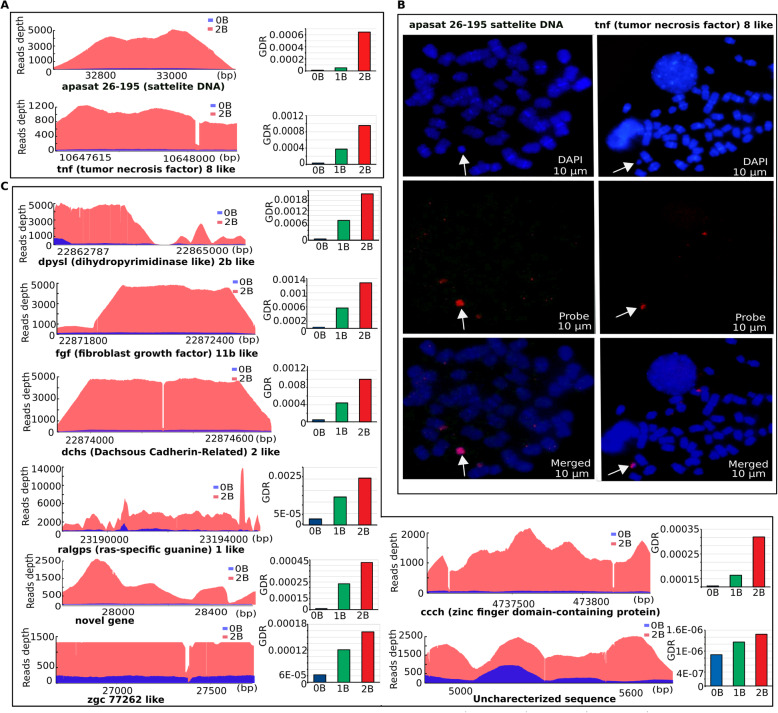
The invasion of amplicon sequences on the micro B of *A. mexicanus* experimentally confirmed using qPCR and FISH. **a** Coverage plots of apa-sat 26–129 satellite and tnf-8 like gene along with the respective GDR qPCR results comparison between B- and B+ genomes. The higher coverage and GDR in B+ genome indicates the duplicated copies of these sequences on the B chromosome. **b** FISH mapping further validated these sequences and showed specific marks (red) in the micro B (white arrows). The metaphasic chromosomes are counterstained with DAPI (blue). **c** Coverage plots of representative B blocks on micro B with corresponding GDR of qPCR. The BLASTn alignments of these representative blocks to Ensemble annotation databases, resulted in several overlapping genes, such as tnf-8 (function: cell death), dpysl2b (function: microtubules binding activity), fgf11b (function: development, morphogenesis, mitogenic and cell survival activities), Zinc finger BED domain daysleeper like (function: chromatin remodelling), zgc:77262 (function: mRNA splicing) ralgps1 (function: cytoskeleton organization) and dchs (function: cell adhesion)

To investigate the abundance of sequences in B+ genomes and to validate coverage-based identification of B chromosome sequences, we used qPCR for relative copy number quantification of 10 randomly selected B blocks in *A. mexicanus* with 0B, 1B and 2B genomes. The GDR values were determined using qPCR results. Higher GDR in 2B and 1B genomes as compared to 0B was confirmed for all the total 10 representative blocks that were selected for this analyses, thus confirming our NGS analysis and coverage approach (Fig. 5; Supplementary dataset 5). In addition, fluorescence in-situ hybridization (FISH) of the two selected B-blocks further confirmed their abundance on the micro B (Fig. 5). The FISH showed specific concentrated signals on the B and some subtle small hybridization signals on a few A chromosomes. The coverage abundance, qPCR and FISH results indicate a strong correlation between NGS and experimental approaches and validate that these sequences are highly amplified on the B of *A. mexicanus.* These two FISH-mapped sequences were annotated as apa-sat 26–129 satellite and tnf-8 like gene that appear to be highly duplicated, suggesting an invasion of these sequences have occurred on the micro B.

To further investigate the B blocks chromosomal organization, we also performed double FISH mapping of randomly selected blocks in *A. flavolineata.* Although these blocks did not evidence any B-specific abundance, the A and X chromosomes showed distinct hybridization marks, mostly clustered in telomeric regions (Supplementary Fig. S10). However, relative less abundant and scattered signals were observed on the B chromosome*,* for certain candidate blocks. The mapped candidate sequences were searched against the ‘nr’ database, and no similarity was found with any gene or repeat element, and were assumed as unknown/uncharacterized genomic regions.

### A majority of B-localized sequences indicate a high level of methylated Cs within CpGs of the cavefish genome

The GO enrichment analysis of cavefish B-localized genes indicated the term “methylation” was among one of the most enriched terms (Fig. 2b). Therefore, we took advantage of the available bi-sulphite sequencing data in NCBI/SRA database of the cavefish to call methylation of the B chromosome sequences. To analyze the methylation level of the micro B sequences in the cavefish genome, we mapped the bi-sulphite treated reads to the B-blocks, which were sequenced previously by Gore et al. [45]. The Bismark mapping of bisulphite Illumina reads to the B blocks of the cavefish yielded a total of 79,288,266 methylation call strings with a total of 32% mapping efficiency. The methylated Cs in the CpG context was 52.1%, remarkably higher as compared to the methylated Cs in the non-CpGs context, which was only 16.2% (Supplementary Fig. S11). These data show that the Cs of B chromosomal sequences are hypermethylated within CpGs regions. The original bottom strand (OB) alignments show that out of a total of 18,340 B blocks, there were 6035 blocks with more than 50% methylated Cs within the CpG context. In contrast, 7560 B blocks were found with less than 50% methylated Cs and the remaining 4745 blocks were unmethylated. Out of 6035 blocks (> 50% methylated Cs), there were 774 B blocks that reported a high level (> 90%) of methylated Cs, suggesting these B-localized sequences might have been repressed or down-regulated due to hypermethylation (Supplementary dataset 6). We further detected a total of 722 CpG islands in the microB sequences.

### Repeatome landscapes indicate an abundance of LTRs and DNA transposons on the B chromosome of *Astyanax*

**Fig. 6:**
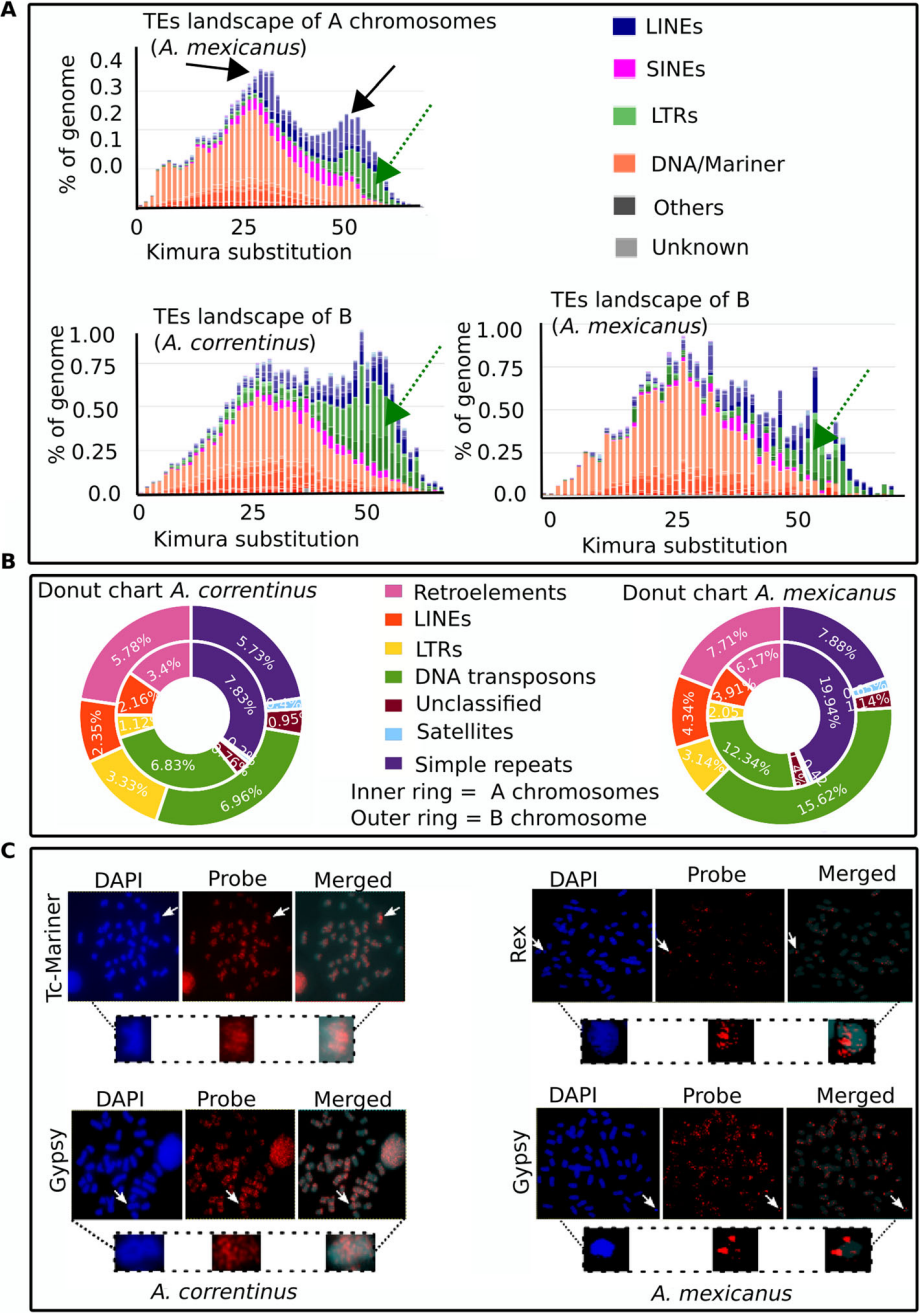
Comparative analyses of TE composition between A and B chromosomes. **a** Comparison of repeat landscapes of TEs provide insights on their evolutionary history in both A genome and the micro B and macro B of *A. mexicanus* and *A. correntinus*. The X-axis shows the percent of TEs in the genome while Y-axis represents the Kimura distances that ranged from value 0, representing recent TE copies, to 50 for the old TE insertions. Black arrows indicate the recent wave of transpositions in the genome of Astyanax genus (black arrows point to transposition waves). The higher abundance of LTRs (green) and other retroelements (blue) in the B chromosome landscapes can also be observed. Green arrows point towards the difference between abundance of A and B chromosome LTRs. **b**. Donut charts show the comparison of repeat composition between the As and B. The outer and inner rings depict A and B chromosomes respectively. Again, the higher percentage of LTRs and retroelements confirm their relative abundance on the B as compared to A chromosomes. Noticeably, the simple repeats percentage was higher on the Bs. **c**. FISH of representative elements on metaphase chromosomes of *A. mexicanus* and *A. correntinus* with B chromosomes analyzed for the organization of Tc Mariner, Gypsy and Rex elements. A dispersed pattern among diverse chromosomes, including Bs, was observed. Magnified view of B chromosomes is shown with the presence of markings of corresponding elements. The abundant signals of these TEs are indicative of their copious nature in Astyanax genome and parallel with the landscapes analyses

We performed a comparative analysis to investigate the relative TE abundance and detect any possible differences in their contents between the regular A chromosomes and B chromosomes in *Astyanax* species. Interestingly, we found that the Bs recorded a higher percentage of TEs, DNA transposons and especially LTR elements as compared to the A genome (Fig. 6a, b). The repeat landscapes of both A and B chromosomes have a larger amount of DNA transposons and LTRs insertions (Fig. 6) reflecting a wave of transposition has occurred during the genome evolution of *Astyanax*. Remarkably, the FISH mapping of some these (representative) elements confirmed the repeatome landscapes analysis (Fig. 6c). We found the typical dispersed signals of hybridization for the respective FISH probes of Gypsy and rex (retrotransposons) and Tc-Mariner (DNA transposon) on the B chromosomes (Fig. 6c). Both FISH and bioinformatics analyses showed that these elements are scattered throughout the genome of *Astyanax*. These elements appear widely distributed throughout all chromosomes with some specific concentrations on certain regions. The wide distribution of these elements across all chromosomes indicates a series of transposition events occurred during the karyotype evolution of *Astyanax*. In addition to TEs, the Bs contain a remarkably higher proportion of simple repeats as compared to As (Fig. 6b).

We also analyzed rDNA clusters by FISH to visualize rDNA organization in the *A. mexicanus* genome. The NGS annotation of B-blocks in *A. mexicanus* did not reveal any 45S rDNA clusters, indicating an absence of 45S rDNA on the micro B chromosomes. FISH confirmed the absence of signals on the micro B as indicated from NGS data (Supplementary Fig. S12). However, we identified eight sites of rDNA clusters on the A chromosomes, with a preferential distribution to terminal portion of the short arms of resident chromosomes.

### Comparative genomics analysis deciphers rearrangements and sheds light on the evolution of B chromosome

**Fig. 7:**
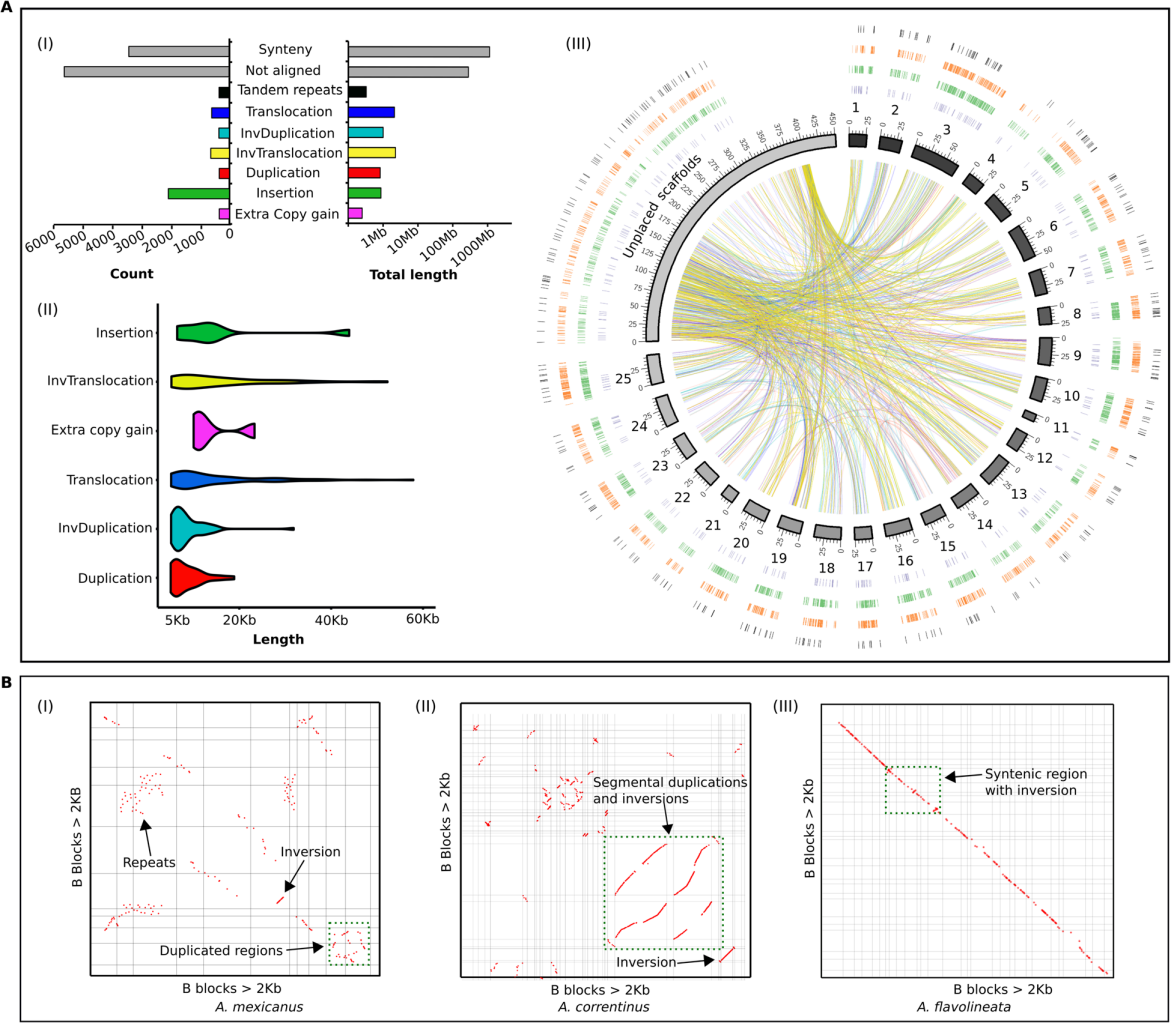
Genomic differences between the B- and B+ genomes of cavefish and B chromosome associated patterns of evolution. **a** (I) The bar graphs show the total number of different rearrangements and their total length in Mb in the B+ genome of *A. mexicanus*. (II) The violin plot depicts the length distribution of each rearranged block in the B+ genome. The distribution of rearrangements with at least 5Kb length is plotted. Refer to Supplementary Fig. S13 for distribution of smaller size rearrangements. (III) Circos plot of B+ genome visualize the different types of genomic rearrangements. Chromosomes (black to grey) are plotted from 1 to 25 with unplaced scaffolds merged as pseudo-scaffolds. The outer rings correspond to tandem repeats (black), deletions (orange), insertions (green), extra copy gains (purple). The inside links show the rearrangements as duplication (red lines), translocation (blue lines), inverted duplication (light blue) and inverted translocation (yellow). **b** The self syntenic dotplots of B chromosomes in *A. mexicanus* (I), *A. correntinus*, (II) and *A. flavolineata* (III) are annotated with different evolutionary events indicated with arrows and green dotted boxes. The dotplot graphics are visualized as filtered “legacy version” whereas the raw images are given as Supplementary Figs. S15, S16, S17

Using a reference-guided approach, we successfully anchored our short read B- and B+ Illumina assemblies of *A. mexicanus* and *A. correntinus* into chromosomes and performed a comparative genomics analysis of these genomes. The whole genome alignments of B- and B+ assemblies and the use of syRI software [46] identified a total of around 6.3 Mb rearranged sequences in the B+ genome including various types of genomic rearrangements such as duplication, inversions, translocation, insertions, extra copies gain and tandem repeats (Fig. 7a; Fig. S13). We detected that a total of 1.13 Gb (87%) of both genomes shared synteny regions with each other. In addition, a considerable amount of these rearrangements were detected within the unplaced scaffolds (Fig. 7 III, circos plot), which represent a majority of the B chromosome sequences. The de novo B+ assembly of the cavefish genome was mapped with the repeat masked assembly representing only coding sequences to reveal the synteny patterns (Supplementary Fig. S14).

We further traced the B associated patterns indicative of duplication and inversions through self-aligned syntenic dotplot analysis generated from the comparative analysis of B blocks of the three species (Fig. 7b; Supplementary Figs. S15, S16, S17). A close view at the syntenic dotplot shows that there is an overlap of the lines when the lines are projected to one axis or the other. The patterns of segmental duplications and inversions as visualized in these dotplots suggest the possible chromosomal rearrangements that might be the main evolutionary forces to derive the B chromosome sequences.

### The B chromosomes of multiple species exhibit similar functional behavior but different genetic contents

A total of 87,6260, 765,254, 69,279,084, 938,990, 2,630,300 and 1,590,728 raw reads previously generated by Illumina next generation sequencing were retrieved from NCBI/SRA database B1 (accession ID: SRX2041358) and B2 (accession ID: SRX2041352) of *L. calcarifer,* B3 (accession ID: SRX1484569) of *E. plorans,* B4 (accession ID: SRX3412298) and B5 (accession ID: SRX3412297) of *A. peninsulae,* and B6 (accession ID: SRX3412293-SRX3412299) of *A. flavicollis* respectively. A total number of 78,611, 65,780, 67,469,719, 660,346, 113,844 and 783,058 decontaminated trimmed reads for the aforementioned respective Bs were aligned with their reference genomes (Supplementary Table S3). The pseudo scaffolding based strategy for assembling these chromosomes with a spacer length 10 kb was considered for annotation and gene ontology analysis.

**Fig. 8:**
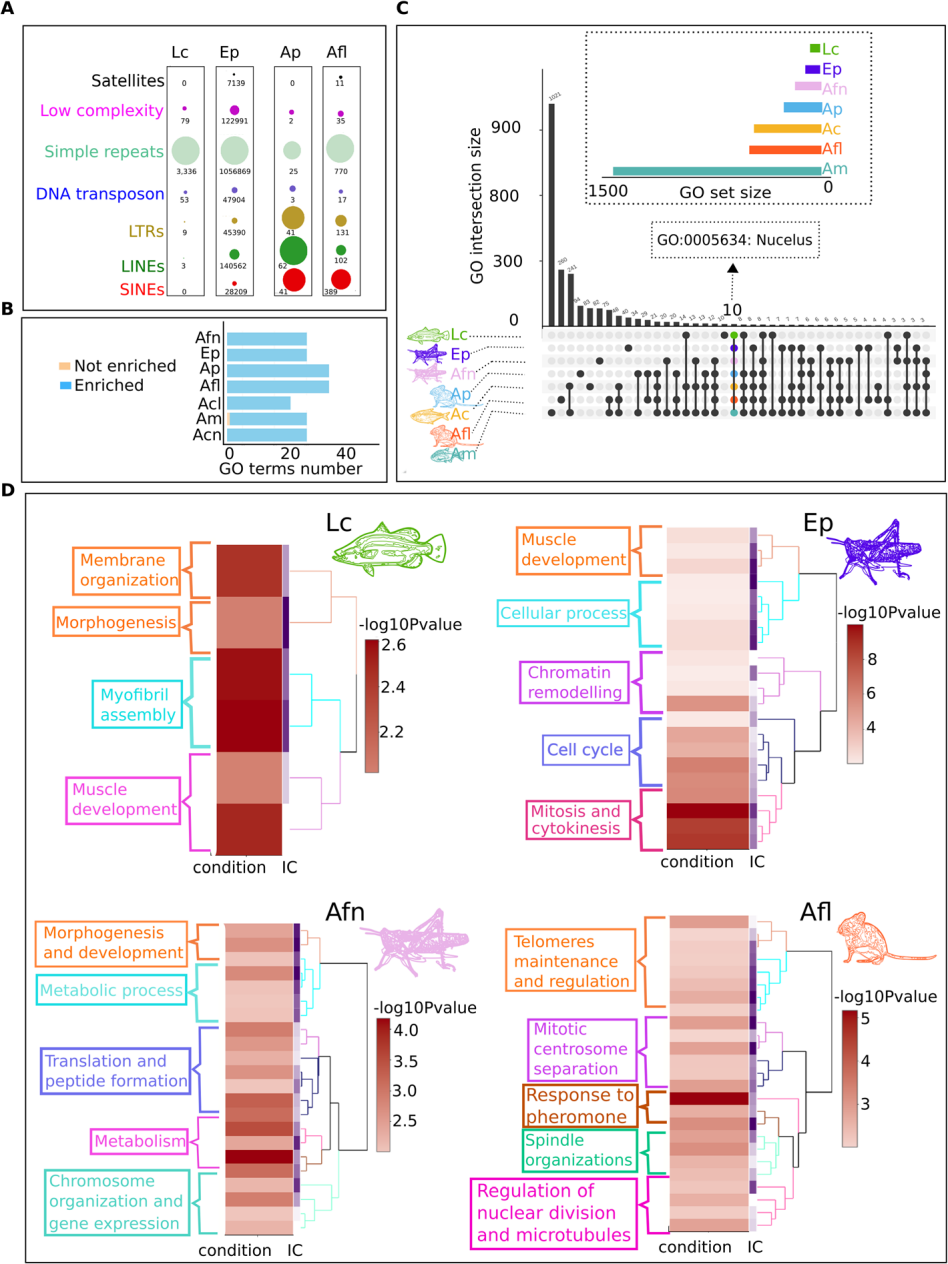
Gene ontology enrichments and comparison of B chromosome functions across all seven species analyzed including the micro dissected sequences (Supplementary dataset 7). **a** Repeat contents comparison of analyzed micro dissected B chromosomes from diverse species. The bubble charts have been merged for all Bs showing the type of content in different colors. Each bubble is a repeat type while each bar indicates a species. The differences between repeats abundance among species suggest that amount of these elements in Bs is subject to their abundance in A genome dependent of species. For example, the Bs of mouse species (B4, B5 and B5) have acquired a higher amount of retrotransposons SINEs, LINEs and LTRs as depicted by yellow, green and red bubbles and lack abundance of satellite DNA. While on other hand, grasshopper Bs (B3) gained a considerable amount of satellite DNA apart from the domination of simple repeats and other elements. **b** Bar chart show the number of enriched and not enriched functions on the Bs of each species after the Fisher exact test. **c** Upset plot represent the comparison of GO among the Bs of all organisms analyzed in this study. A total of 10 GO are shared across all studied species (Table S4). An arrow points to an important GO term “nucleus” that is common among all Bs. The Y-axis corresponds to GO intersection size while X-axis represents the unique and shared GO terms. **d** Enrichment clustering heatmap plots are given for the micro dissected Bs as well as high confident genes (log2 ratio > 2) detected on the B of *A. flavolineata.* The abbreviation Lc, Ep, Afn, Ap, Afl, Ac, Am refers to *L. calcarifer, E. plorans, A. flavolineata, A. peninsulae, A. flavicollis, A. correntinus* and *A. mexicanus* respectively

Although the NGS data of micro-dissected Bs does not cover the complete sequences, we present an estimated and preliminary assembly and annotation of their genes and repeat contents. The repeat annotations of these Bs showed that they have different levels of each repeat across different species (Fig. 8a). The Bs of fish species (B1 and B2) are mainly comprised of simple repeats. Other repeat types such as low complexity and DNA transposons were also detected in abundance for Bs of fish but lacking SINEs and satellites. Similarly, the Bs of grasshopper (B3 of *E. plorans*) were also enriched with simple repeats but notably, the second highest number of repeats sequences were retroelements (SINEs and LINEs), which were not abundant in B1 and B2 of fish. On other hand, the Bs of *Apodemus* species (B4, B5 and B6) contained an abundance of SINEs and LINEs but lack the amount of satellite sequences, which are found in higher number in grasshopper species. The gene annotation recorded for all microdissected Bs revealed several genes overlapping with their reference annotations. Due to the low coverage of sequencing data, the number of genes in micro dissected B sequences can be underrepresented. Interestingly, the GO enrichment analysis of the Bs in different organisms shared some common over represented functions such as metabolism, development and morphogenesis (Fig. 8b, c, d; Supplementary Table S4; Supplementary dataset 7). Moreover, the Bs of these organisms might exhibit a similar functional behavior, for instance the enriched functions like cell cycle, mitosis, chromosome organizations, telomere maintenance, microtubules and spindle organizations (Fig. 8d). These results highlight the importance of genes associated with these functions in the evolutionary survival of the B inside the cell.

## Discussion

The current work demonstrates a high throughput genomic analysis of B chromosomes in two candidate vertebrates and one invertebrate species as well as the microdissected Bs sequences of diverse organisms. We present a comprehensive analysis of *A. correntinus*, *A. mexicanus* and *A. flavolineata* genomes for both B+ and B- individuals with the aim of unveiling the genomic composition, structure, functional and evolutionary dynamics of B chromosomes in these species. Applying a comparative coverage technique, we detected a total of 43.82 Mb and 15.41 Mb of different A chromosomes of *A. correntinus* and *A. mexicanus* respectively, that has contributed to the B chromosomes composition. We found that at least 246 Kb and 58 Kb of unique sequences are exclusive of the Bs in *A. correntinus* and *A. mexicanus* respectively, that do not occur on the regular A genomes. These NGS results are parallel to the size of the corresponding macro and micro Bs as were observed in their karyotypes, demonstrating that the coverage-based approach was successful in deciphering a considerable amount of sequences on the Bs. Our characterization and annotation of B blocks in *Astyanax* species featured a higher amount of gene content and the number of blocks for *A. correntinus* as compared to *A. mexicanus* which is also in agreement with karyotype data. The number of detected blocks of *A. flavolineata* was underrepresented due to low overall coverage of reads obtained as compared to its giant genome. Nevertheless, we were able to detect at least 2.05 Mb of A chromosomal DNA copied into its B, with 194 Kb of B-exclusive sequences.

To perform a deep survey of DNA repeats, we applied a combination of approaches to predict major TEs and their abundance in the genomes and to perform comparisons of the B+ and B- repeat content. Our repeat analysis showed that *A. correntinus* and *A. mexicanus* genomes are comprised of 66 and 35% repeats, respectively, with a domination by DNA transposons, which is comparable to most published fish genomes [47–52]. We highlighted the major repetitive contents of the Bs and our analysis identified TEs, including retroelements, of the B chromosomes of multiple species. Several studies have previously found that Bs are generally enriched with TEs [4, 24, 33, 53–59], suggesting that TEs are the principal migrants of the Bs that may be key players in the insertion of other sequences from As into B chromosomes during evolution. We found a high enrichment for the GO term “transposition” (Fig. 2b) in the Bs of *A. mexicanus* and *A. correntinus*, providing evidence to support this hypothesis. The high level of gene fragments on the Bs (with < 50% integrity) indicate that genic sequences might have been either inserted as fragments, or broken during migration from As into B chromosomes as a result of series of transposition events. Although the microdissected B chromosomes sequences data was not sufficient to draw a conclusion about their repeat contents, the comparative analyses provide an overview to hypothesize that the B chromosomes repeat contents can vary among different species depending upon the repeat content and abundance within the A genome.

The annotation of B-blocks revealed that the Bs contain many gene-like sequences. Our integrity analysis showed that Bs contain many fragmented genes which are possibly pseudogenes and might have formed from their parental genes on A chromosomes during their incorporation into B chromosomes. These putative pseudogenes may have lost their functional ability after duplication from the parental A genes. However, previous work [60] reported that the B of rye harbors pseudogene-like fragments, which are expressed in a tissue specific manner and thus might retain function.

In addition to the fragmented genes, there are complete genes that have remained intact, possibly due to their role in the evolutionary survival of the B chromosome. *These findings support the emerging hypothesis reporting B-localized genes* [36, 37] *according to which B chromosomes accumulate cell cycle genes that might play an important role in their transmission.* Table 2 lists integral B-localized genes in multiple species that are directly involved in cell cycle regulation and chromosome organization processes, including proteins coding for a variety of functions such as chromosome segregation, spindle fibers, microtubules, chromatin organization, chromosome condensation and regulation of the cell cycle. The enrichment of genes associated to cell cycle and chromosomes functions on both microB and macroB of *Astyanax*, suggests that independent of evolutionary stages of B, the gains of such genes benefits its transmission, which further reflects its selfish behavior. Remarkably, the GO enrichment analyses of different micro dissected Bs in different species revealed similar patterns of functions, thus providing evidence to corroborate the emerging hypothesis that the evolutionary success of the B chromosome lies on its gene contents. Enrichment analysis detected diverse GO terms for important biological roles such as metabolism, cell adhesions, reproduction, stimulus response, localization, morphogenesis and methylation. Genes with such functions were also reported to be located on B chromosomes in previous studies (see a comprehensive and updated list of B genes in the review by Ahmad and Martins [2]*).* Among the B enriched functions, we found that most of them are involved in developmental processes, particularly morphogenesis. The gene indian hedgehog b (*ihhb*), involved in morphogenesis, was previously identified as highly duplicated on the B chromosome of the cichlid fishes [61, 62]. These commonly enriched functions shared among the Bs of different species suggest that B chromosomes exhibit a conserved behavior to acquire a certain role, although their genetic makeup in may vary across different taxa. Notably, the higher level of metabolism related B-genes in *A. mexicanus* are interesting because this fish species has been reported to have a more efficient metabolism as compared to other fishes [63]. The cavefish has adapted evolutionary traits to tackle the scarcity of food and the cave environment. Of these traits, the adaptation to evolve sensitive mechanosensory organs and chemical senses are the significant compensatory changes due to possibly strong selective pressures. The GO enrichment of such functions on the B of cavefish offer exciting insights into whether B chromosome provide extra genomic compartments for the evolutionary success of this species, suggesting that Bs might have played some role in shaping the genome evolution for effective adaptation in cave environment. Metabolism related genes have been found on the Bs of cichlids [2, 36] and interestingly these fishes use mechanosensory receptors mainly for mating and species recognition, and consequently, specific metabolism is required. We therefore hypothesize that the B chromosomes plays a role in adaptation acting on metabolisms.

Besides the genes discussed above, there are enriched genes related to reproduction detected on the Bs of *A. correntinus*, suggesting that Bs can also have a functional impact on sex determination, as previously described by Yoshida et al. [61] in cichlids. Our karyotype data of around 60 individuals in *A. mexicanus*, revealing male-specificity of the B chromosome, also point towards this phenomenon that the presence of Bs may have some role to determine the sex in *Astyanax* [64].

The BLAST results of B chromosomes exclusive sequences did not revealed any significant homology with ‘nr’ database, thus indicating their novel and unique nature. However the similarity of few weak hits to mitochondrial genes shows that such sequences sourced from mitochondria, might have been inserted into B of the *A. flavolineata* and subsequently might have evolved into novel sequences. The mitochondrial gene *MTG1* (mitochondrial GTPase 1) has been recently reported on the B of another grasshopper, *E. plorans* [37]*.* Previously*,* mitochondrial sequences and B chromosomes in grasshoppers have been reported for their role in the substitution and variations among different populations [65]. Most (more than 90%) of the exclusive B chromosome sequences of *Astyanax* were characterized as novel and unknown genes as well as some long non-coding RNA sequences.

The epigenomic profiles of the microB in the cavefish genome provide a rough view of the methylation status of B chromosome sequences. Although our data analysis supports that most of the B chromosome sequences in the cavefish are likely methylated mainly within CpG regions, it still remains to be seen whether there is any methylation based repression of any gene and whether these epigenetic changes might have any phenotypic variability effect. Furthermore, since methylation patterns are often tissue specific to effect the regulation of genes, a comparative analysis of these sequences between B+ and B- from the same tissue types would be much more informative to obtain clear profiles of differentially methylated and expressed regions, and explain the impact of B chromosomes in this context. Taken together our analysis, suggest that DNA methylation of B chromosome sequences might be one of the principal mechanisms to mediate the repression of many B-localized genes and prevent any further phenotype impact that might have happened due to the occurrence of micro Bs in the cavefish genome.

**Fig. 9:**
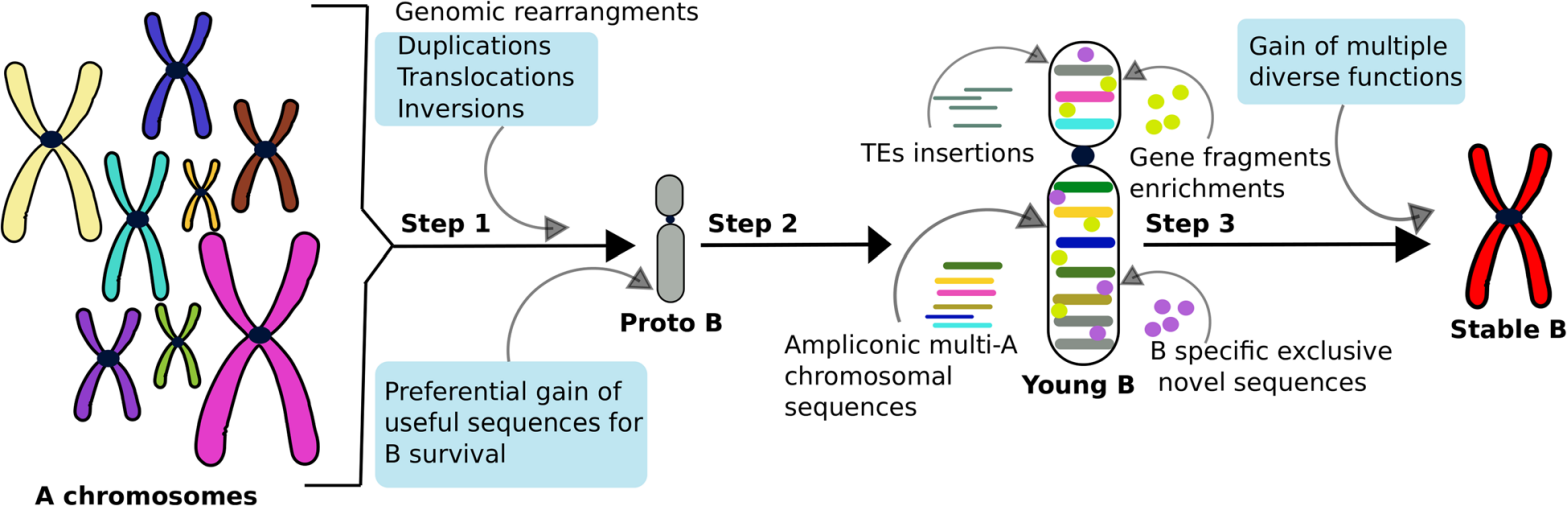
A schematic view of B chromosome evolution. During the first step, a proto-B is derived from multi-A sequences as a result of genomic rearrangements. The proto-B gains the sequences from A genome for its survival and successful transmission. In the second step, the proto-B accumulates further sequences with a series of TE insertions, ampliconic sequences, gene like fragments and the formation of unique sequences that are specific to the B. Finally, a mature B evolves, providing extra genomic material which may contains genes for diverse functions

The rearrangements analysis of the B- versus B+ genomes suggests that the cavefish genome exhibits extensive rearrangements that might have shaped its extra-ordinary evolution for adaptation. Moreover, the comparison of the B blocks to their ancestral A genome regions allowed us to infer the evolutionary mechanism that led to Bs. The homology of B sequences to different A chromosomal sequences indicates that after the proto B was formed, it might have gained sequences from across the rest of the genome and subsequently experienced duplications and rearrangements. An emerging model proposes that the B sequences are most likely inserted through subsequent transposition, duplication and rearrangement events to form the B chromosome (see an evolutionary model as Fig. 9). The different sizes of the B blocks ranging from few hundred to thousands bp indicate that the larger regions might have migrated to Bs after the formation of a proto B. The abundance of TEs in these blocks suggests that transposition facilitated the movement and incorporation of these sequences, followed by duplications events as detected in our analyses.

Although the identification of both fragmented and complete genes on the Bs provides interesting insights, it remains unknown whether these genes are active. While we predict most of the fragments are pseudogenes, further analysis of transcription levels will assist in understanding exact structure and function of these gene fragments. It is possible that the enriched fragmented genes on B chromosomes may represent gene fusions, and thus may be transcriptionally active but could have altered functions from their progenitor genes. Furthermore, actively transcribed fragments from these truncated partial genes may have some function in regulating the activity of other genes through interference. The transcriptional activity of B-located genes involved in cell cycle has been found in a grasshopper species, *E. plorans* [37]. While there are few other studies that have confirmed the transcription expression of B-located genes [38, 42, 60, 66], an analysis to test the function of our B detected sequences will serve to find out the active genes playing a role in controlling B chromosome behaviors such as sex bias and drive. A better understanding of the structure and function of the B can be achieved by a complete high-quality B chromosome assembly, that will be a priority step in future for B chromosome researchers.

## Conclusions

This paper offers contributions about the genomic composition, evolutionary and functional aspects of multiple B chromosomes in different species. Applying a coverage based comparative approach we detected a considerable amount of B chromosome segments that contain many gene fragments, few complete genes and an abundance of TEs along with other repeat types. We revealed that the B-localized genes are associated with diverse functions, some of which may explain the evolutionary fate of B chromosome. We also found patterns of genomic evolution such as duplications and rearrangements events that might have shaped the evolution of B chromosomes. Taken together, we conclude that the Bs, which were believed for a long time to be inert elements, may in fact participate in relevant genome function and evolution. Our present research opens new avenues and interesting prospects for future research and therefore encourages further studies to investigate the expression of detected B-localized genes to decipher their role in a myriad of cell processes.

## Methods

An overview of the methodology is illustrated in Supplementary Fig. S1.

### Model organisms and karyotyping

We obtained the specimens of *A. mexicanus* (cavefish) from a local fish store in Botucatu, Sao Paulo, Brazil. All the cavefish animals used in the present study were sourced from the same commercial company, namely “Aquarismo Aquamundi Botucatu”. These specimens were then maintained for further karyotype analysis in the fish facility of Integrative Genomics Laboratory of UNESP - Sao Paulo State University. The specimens of *A. correntinus* were collected from its natural habitat in the Iguaçu River, in the stretch with around 25 km between downstream of the Iguaçu Falls and its mouth on the Paraná River, Brazil. All the *A. correntinus* specimens used in the present research were obtained with the permission and ethical approval of Western Paraná State University (UNIOESTE). The grasshopper *A. flavolineata* specimens were obtained from their natural habitat in Rio Claro, Sao Paulo, Brazil. The experiments involving all animals were performed according to agreement of ethics set by Brazilian College of Animal Experimentation and the use of specimens in the experimental work were approved by ethical committees of Institute of Biosciences/Unesp (Protocol no. 769–2015) and CEEAAP/Unioeste (Protocol 13/09). A total of 129 animals were used in the present study including 21 *A. correntinus*, 39 *A. mexicanus* and 69 *A. flavolineata* individuals*.* The total number of animals was required to perform the karyotyping and observing B chromosome occurrence and frequency in individuals. The animal size sample was calculated according to the experiment requirements: to determine the B carrying individuals; raise samples with chromosome quality for karyotyping and FISH mapping; to determine the ration of 0B, 1B and 2B individuals; comparative male and female prevalence of Bs; and genomic DNA extraction for qPCR and genome sequencing.

All animals were euthanized in order to be dissected and extract tissues for chromosomes preparation and genomic DNA extraction. The fishes were submitted to euthanasia by immersion in eugenol 1% anesthesic for three minutes. The grasshoppers were anesthetized with ethyl ether for about 10 min. The chromosome preparations of *Astyanax* fishes were obtained from anterior kidney cells using 0.02% colchicine treatment for 30 to 40 min following the protocol of Sumner [67]. Mitotic chromosomes of *A. flavolineata* were obtained from the embryos. The karyotyping procedure involved classical cytogenetics using a Giemsa stain to identify different chromosomes as metacentric, submetacentric, subtelocentric or acrocentric. Thirty metaphases spreads from each individual were analyzed and ten best mitotic metaphases were used to measure karyotypes for each species. The male individuals were identified and confirmed from histology of testis. Individuals carrying B (B+) and those without B (B-) chromosomes were identified by karyotype analysis. The genomic DNA samples of B- and B+ male individuals were analyzed on agarose gels to verify the integrity and quantified by nanovue spectrophotometer and Qubit Flourometer to obtain the information about concentration as required for sequencing.

### Illumina next-generation sequencing

After performing quality control (QC), qualified DNA samples were processed for library construction. A total of eight samples of all male individuals including 0B (−B) and 1B and 2B (B+) of model organisms were sequenced using HiSeq Illumina (Table 1). Genomic DNA of all eight samples was randomly fragmented to prepare sequencing libraries followed by 5′ and 3′ adapter ligation. For each individual sample, a separate set of libraries were constructed and paired end sequencing was performed with the read length of 151 bp. Raw data from Illumina’s HiSeq machine was processed with Illumina software to generate Fastq files. The Illumina reads were screened for sequence quality using FASTx toolkit [68], low quality reads were discarded, and adapters were trimmed using the Trimmomatic tool [69]. Specific filtering parameters were set in the commands according to requirements for removing adapters and poor quality reads as per the FastQC tool [70] report. Filtering of reads was performed by FASTx toolkit using parameters set to quality number 28 and percentage value 80 for alignments.

### Genome alignments and de novo chromosome scale reference-guided assemblies

The chromosome scale assembly of *A. mexicanus* genome [45] was used as the reference genome for alignments of *A. mexicanus* sequencing datasets. The genome assembly of *A. mexicanus*-2.0 (GCA_000372685.2) was downloaded from Ensembl (https://www.ensembl.org/Astyanax_mexicanus/Info/Annotation) [71] and used as a reference genome for alignments of *A. mexicanus* B+ and B- reads. To create assembly references of genomes containing B chromosomes, we assembled the Illumina reads of the B+ samples for *A. mexicanus*, *A. correntinus* and *A. flavolineata* using SOAPdenovo [72]. We determined different K-mer size based upon the read length, sequencing depth, the total genome size and the computer memory for each size for each of the species. We performed several trial runs of the assembly and chose the best Kmer values (93 for *A. mexicanus*, 63 for *A. correntinus* and *A. flavolineata*), which yielded the maximum N50 and N90 in the finalized assemblies. We mapped the filtered Illumina B- and B+ genomic reads against their reference assemblies using the “very sensitive” parameter of Bowtie2 [73]. We further *de novo* assembled the B+ and B- (short reads assemblies) genomes separately for *A. correntinus* and *A. mexicanus* and anchored the scaffolds into chromosomes (linkage groups) by Ragoo assembler [74] using the retrieved chromosome level assembly of *A. mexicanus* as the reference genome*.* The evaluation of the *de novo* assemblies was obtained using QUAST software [75] by computing several metric values (length, number, length variation, N50, gap length). Refer to Supplementary Note 1 for more details (Supplementary Table S1).

### Coverage analysis and identification of B chromosome regions

The identification of sequences present on B chromosomes was performed using statistical parameters of aligned reads coverage comparison between B+ and B- genomes, as proposed by Valente et al. [36] with modifications to improve the analysis. The sites with at least 15× reads coverage in B+ and B- genomes were selected, and the per-base coverage of the B+ and B- genomes were investigated using bedtools [76] to measure the mean B+/B- coverage ratio (MC). Then, normalized coverage (NC) was obtained as: $\mathbf{NC}=\frac{\mathbf{Raw}\kern0.5em \mathbf{coverage}}{\frac{\mathbf{Region}\kern0.5em \mathbf{size}}{\mathbf{MC}}}$. The mean ratio (MR) and standard deviation (MRSD) for the genome region with most similar size and raw coverage, not containing B sequences, were obtained with NC. Next, the B+/B- regions with coverage < $\frac{\mathbf{MC}}{\mathbf{2}}$ were removed and B+ ratio (BPR) was calculated with *NC* $\frac{\mathbf{B}+}{\mathbf{NC}\kern0.5em \mathbf{B}-}$. Regions with BPR **≥MR +** (**SD × N**) were selected, were N is the number of SD required to determine a block. This way, we were able to set an estimated threshold for detecting the extra copy of A chromosome sequences in a B+ genome which can be regarded as putative B chromosomal sequences, known as “B-blocks”. Our custom python script for identification of these blocks is available online at github (https://github.com/farhan-phd/Integrative-genomic-analysis-reveals-the-gene-contents-repeats-landscapes-and-evolutionary-dynamics/tree/master). The B-blocks were constructed using two levels (200 bp and 1 kb) of tolerances; for example a level of 200 bp means that the B sequences within 200 bp regions and that have mean ratio greater than, or equal to, the established value can be considered one part of the same block. In this way, four different sets of B-blocks (0 stdv and plus 2 stdv, both with tolerance of 200 bp and 1 kb) were obtained. Finally, the blocks with 2 stdv and 200 bp, the most stringent conditions, were considered for further analysis. The B-blocks were manually visualized using J-browser [77] and comparative plots were created using the “Sushi” package [78] of Bioconductor (https://www.bioconductor.org/) pipelines in R.

In addition to the coverage ratio analysis, we also isolated the sequences that were located exclusively on the B chromosome and completely absent on the As. We screened the B containing genome and analyzed the read alignments in a way that a minimum of 200 bp region does not concede any B- alignments at all, whereas at least 50× coverage B+ alignments will have continuous and uninterrupted reads mapped at the same region. The complete absence of B- alignments means that the respective region is missing in the 0B genome (A chromosomes) and due to significant representation of the B+ reads aligned to this region(s), this sequence is potentially specific to the B chromosome. The reads alignments were measured for each exclusive B sequence of all B- and B+ genomes with the Bedtools coverage pipeline. The fasta sequences of B blocks and exclusive regions have been provided as Supplementary datasets 8, 9, 10, 11, 12, 13.

### Search of protein coding sequences on Bs

To screen the B chromosomes of our model species for protein coding sequences, we employed a similar approach as Navarro-Domínguez et al. [37]. The transcriptome assemblies of *A. mexicanus* (used as reference for *A. mexicanus* and *A. correntinus*) and *Locust migratoria* (used as reference for *A. flavolineata*) were retrieved from NCBI database (accession IDs: GDIO00000000.1, PRJNA237016). Against these reference transcriptomes, we mapped the reads obtained from the B- and B+ genomes, using “local sensitive” parameters of Bowtie2. We calculated the total quantity of reads that aligned for each transcript and compared the difference between the respective abundance of B+ and B- reads using an available python script published by Navarro-Domínguez et al. [37] (https://github.com/fjruizruano/ngs-protocols/blob/master/count_reads_bam.py). The putative B-located coding sequences (CDS) were identified on the log_2_(B+/B-) ratio considering a minimum of 40 aligned reads to each contig. Thus, the transcript for which a log_2_ ratio equal to or greater than one was assumed as a B representative sequence. For instance, a single copy B-located sequence, which represents two copies in a 0B diploid genome, will have an extra third copy in 1B genome due to the B chromosome. Whereas a 2B genome will have two extra copies, therefore the log_2_(2) = 1, so we chose to use this value as threshold to extract the B-localized CDS.

### Repeats and genes annotation of B chromosome blocks

*The B-blocks and B-located CDS* were first annotated for repetitive DNAs using RepeatMasker 4.0.3 [79]. The repeats were masked using the reference database of metazoa. We assayed for under-representation of TE super-families using the equation: TE = % of TE family in the genome × 100 / total repeats content in the genome, as described by Mcgaugh et al. [47]. We also performed a comparative repeats composition analysis between A and B chromosomes. Results were parsed by Perl script to depict the relative abundance of repeat classes using the RepeatMasker outfiles. The repeat landscapes were generated with the RepeatMasker “calcDivergence- FromAlign.pl” and “createRepeatLandscape.pl” utility scripts.

We also annotated B-blocks and B-located CDS to search for genes by comparing them to the reference gene sets of close species downloaded from NCBI databases. The reference gene sets consists of *A. mexicanus* for *Astyanax* species and *Drosophila melanogaster* for *A. flavolineata.* These references were selected on the basis of the complete representation of genes and high-quality chromosome level assembly. We calculated the integrity score (0–100%) and gene length of all B-genes found in the B-blocks by combining all DNA pieces related to the same gene (each “piece” is a different gene length in each list of blocks). Blocks with integrity scores < 50% mean that they are highly fragmented or incomplete genes. The higher integrity score, the more probable intactness of genes is expected. The identity percentage was calculated comparing the length of identified genes on Bs versus the total corresponding gene length in the annotation of the reference genomes. The total gene length was recovered by the sum of all pieces. The integrity percentage of each B-gene was calculated comparing its length to the corresponding gene length in the annotation of the reference genomes. Finally, the integrity percentage for each gene was determined and the genes were categorized in different groups (from 0 to 100%) on the basis of integrity percent. The remaining CDS that were neither aligned to genes nor repeats were termed non-annotated or unknown sequences.

### Quantitative real-time PCR (qPCR) and fluorescent in situ hybridization (FISH)

To validate B sequences identified by bioinformatics analysis, we performed polymerase chains reaction (PCR) and used the amplified PCR products as FISH probes. Primers for selected sequences were designed by NCBI/Primer-Blast (https://www.ncbi.nlm.nih.gov/tools/primer-blast/) and PrimerQuest (https://www.idtdna.com/Primerquest/Home/Index) tools. Primer quality was evaluated by PCR Primer Stats program (http://www.bioinformatics.org/sms2/pcr_primer_stats.html). Primers designed for FISH probes and qPCR experiments for TEs and B blocks of *A. mexicanus* and *A. correntinus* are listed in Supplementary Table S2.

Randomly selected B-block sequences were used to design primers for qPCR to confirm the genomic data and relative abundance on the B chromosome of *A. mexicanus*. Genomic DNA from each of two individuals (total six sample triplicates) containing 0B, 1B and 2B chromosomes were diluted to 40 ng/ μL and used as a template to measure the gene dose by CT method of relative quantification [80]. We selected the *pde4ca* gene as a reference, which resides on A chromosomes and thus has the same copies on both B- and B+ genomes. The gene dosage ratio (GDR) was calculated by comparing the mean CT values of both target sequences (blocks) and reference gene according to Valente et al. [36]. The experiment qPCR was performed on StepOne Real-Time PCR Systems (Life Technologies, Carlsbad, CA) with cycling conditions; 95 °C for 10 min, 45 cycles of 95 °C for 15 s, and 60 °C for 1 min. The dissociation curve was observed to confirm the specific amplification of the PCR products.

The FISH probes were labeled with Digoxigenin-11-dUTP (Sigma) and stringent conditions were applied to perform FISH according to the protocol of Pinkel et al. [81]. Slides were prepared by dropping 10 μL of chromosomes suspensions and subsequently treated with RNAse. Different conditions were optimized for each probe during pre-hybridization washing steps and denaturation of chromosomal DNA was performed in 70% formamide for 15 s at 65 °C. The hybridization mix containing 10% dextran sulphate, 2*×* SSC, 50% formamide and labeled probe was denatured for 15 s at 65 °C and dropped on denatured chromosomes for overnight hybridization at 37 °C. The post-hybridization washing steps were adjusted for each probe (from 3 to 5 min) and detection of probes was performed with digoxigenin-rhodamine (Roche), followed by staining of slides with DAPI (4′,6-diamidino-2-phenylindole, Vector Laboratories). The microscopic examination of the slides was done in an Olympus BX61 optical microscope. Metaphase images were taken on an Olympus DP72 and optimized using GIMP (GNU image manipulation program).

### Sequence analysis of microdissected B chromosomes in multiple species

In addition to the whole genome analysis of our candidate sequenced species, we included four additional species to test our hypothesis about the B chromosome evolution. For these additional species, NGS Illumina data for microdissected B-chromosomes of *Eyprepocnemis plorans* (grasshopper) [82], *Lates calcarifer* (Asian seabass fish) [83], *Apodemus flavicollis* and *Apodemus peninsulae* (mouse) [84] were downloaded from the NCBI-SRA database. We analyzed these Bs because the genomic composition, including genes search, gene ontologies as well as repeat annotation of these microdissected Bs, have not been previously performed. The NGS reads with a quality score < 20 bp were removed using FASTX-Toolkit, adapter sequences and low quality bases were trimmed using the cutadapt pipeline [85] and Trimmomatic [67]. Clean reads were mapped to the respective assembled reference genomes using Bowtie2 with default parameters. The reference genomes consist of *L. calcarifer* [83], *Locust migratoria* [86] and *Mus musculus (GRCm38.p6)* genome [87] assemblies, which were retrieved from the NCBI/Genome database. Successfully mapped reads were chained together across gaps < 10 kb to form B chromosome pseudo-scaffolds. Pseudo-scaffolds were assembled using CAP3 [88] to remove redundancy and the generated contigs were manually checked to reduce potential mis-assemblies. The microdissected B chromosome assemblies were performed on the basis of the pseudo-scaffolding strategy as proposed by Vij et al. [83]. The assembled B microdissected chromosomes were then compared to their reference gene annotation sets to identify their respective gene contents. The reference set of genes was retrieved from the Ensembl browser (https://www.ensembl.org/index.html) and we used BLASTn [89] for homologous gene annotation. The references consisted of *Gasterosteus aculeatus, Drosophila melanogaster,* and *Mus musculus* for B chromosome analyses of *L. calcarifer, E. plorans* and *Apodemus,* respectively, selected on the bases of completeness of gene annotations.

### Functional annotations and gene ontologies enrichment analysis

We used the ViSEAGO package [90] of R (Bioconductor) to perform the following analysis. First we retrieved the list of B chromosome genes for each species from the Blastn output with the best BLAST hit having at least 200 base pairs of the query sequences overlapped with respective reference gene. We then downloaded a complete annotation database of all genes of the reference species from Ensembl. Through ViSEAGO, we conducted a functional genomics analysis of both the single and multiple sets of B chromosome genes against the complete set of reference genes as a background set of genes. The latest version of gene ontologies (GO) databases were loaded in R for each species from Ensembl and functional enrichment analysis were performed using Fisher’s exact test [91].

In this analysis, list of B chromosome genes of each species was compared to the background set of entire set of genes in the reference genome. The background sets were retrieved from Ensembl using Biomart in Bioconductor. GO terms were obtained based upon the *P*-values, which represent the degree of independence between related terms. Tables of results, summarizing the functional enrichment tests, were obtained for each species. The enriched GO were grouped together on the basis of semantic similarity (SS) according to their topological positions and annotations in GO graph. The IC value, which is the negative log probability of occurrence of a GO term was computed and clustering of enriched GO terms was performed using the graph based method of Wang et al. [92]. Both single and comparative heatmap plots were graphed with -log10(*p*-value) from the enrichment statistical tests and IC value of the GO clusters to profile the functional overview and biological interpretations of the B chromosomes. The enriched terms were organized in clusters with respect to their similar topologies and dendrogram in way so that the GO terms share the common functions. A comparison of GO terms across the Bs of multiple species was also performed to reveal the common functions shared among the genes residing on Bs, and the results were plotted as upset graph in R.

### Epigenomics profiling of the B chromosome in *A. mexicanus* using bisulphite sequencing data

The methylation status of the micro-B sequences in *A. mexicanus* was assessed using the whole genome bisulfite sequencing data generated for this species recently by Gore et al. [45]. The data was retrieved from NCBI SRA (accession: GSE109006). This sequencing was performed from the eye tissues of cave fish, *A. mexicanus* on an Illumina HiSeq2500. We trimmed and filtered the low quality reads from the raw sequences using Trimmomatic and considered the high quality reads for alignments. We used the Bismark tool [93] to map the bisulphite reads against our B-blocks as a reference. First the fasta sequences of the B-blocks of *A. mexicanus* were indexed and converted into bisulfite sequences. In the second step, the high quality bisulfite reads were aligned to the B blocks to output the SAM file and methylation call report. In the third step, methylation information was extracted from alignment output by running methylation extractor of the Bismark software.

### Comparative genomics and rearrangements detection

To investigate the genomic differences between the B- and B+ genomes, we performed the whole genome alignments of our *de novo* assemblies using nucmer in MUMer package [94]. Both B+ and B- genomes of *A. mexicanus* were mapped with “--maxmatch, -c, -b, and -l” options of nucmer to balance and resolve alignments. We filtered the alignments with the “delta filter” script of the MUMer and the output filtered files were parsed in the tab delimited files with the “show coords” tool. The B- and B+ assemblies were selected as reference and query genomes, respectively, for the identification of rearrangements. Genomic rearrangements were identified using syRI – Synteny and Rearrangement Identifier [46] with default parameters. The unplaced scaffolds of both assemblies were merged as pseudo-scaffolds and the chromosome IDs were renamed and formatted before syRI. The output files were parsed using custom bash commands and the rearrangements plotted as circos graphics using Clico FS [95]. The bar graphs and violin plot were generated in R. To reveal the homology of the B chromosome sequences of *A. mexicanus* with A chromosomes and identify their ancestral sequences, we compared the B chromosome of *A. mexicanus* to the reference. To find putative regions of homology between ancestral sequences of B blocks, we identified colinear regions of sequence similarity to infer synteny and generated dotplots of the alignments. For this analysis we chose the largest blocks with size greater than 2 kb and did a comparison using using CoGE SynMap [96] to identify the evolutionary genomic patterns of the B chromosome. Different syntenic patterns were interpreted according to the dotplot explanation examples given in the CoGepedia (https://genomevolution.org/wiki/index.php/Syntenic_comparison_of_Arabidopsis_thaliana_and_Arabidopsis_lyrata) [97]. We also compared the B+ *de novo* genome of *A. mexicanus* with the reference hard masked genome containing only CDS to reveal the syntenic patterns.

### Supplementary information

**Additional file 1.** Supplementary Note 1.

**Additional file 2: Table S1.** De novo genome assemblies and their statistics. **Table S2*.*** List of primers of representative blocks used in qPCR and FISH mapping*.* **Table S3.** Summary of analyzed data used for microdissected Bs assemblies. **Table S4.** List of 10 common functions shared among the Bs of all seven analyzed species.

**Additional file 3: Figure S1.** A workflow of steps applied in the present study during the procedure of genomics analyses of B chromosomes in different species. **Figure S2.** Coverage plots of B-blocks of *A. correntinus* with remarkable difference in the reads coverage between 0B and 1B samples. **Figure S3.** Coverage plots of B-blocks of *A. flavolineata* with remarkable difference in the reads coverage between 0B and 2B samples. **Figure S4.** Identification of B chromosome genomic blocks (A) and their repeats contents (B) in *A. flavolineata*. **Figure S5.** Identification of protein-coding genes located in B chromosomes of the *A. mexicanus*, using the number of mapped reads that map to the CDSs found in the transcriptome, in the 0B (X axis) and 2B (Y axis). Each dot represents a coding sequence with only those labeled that recorded the log_2_ greater than 1.5. The plot is limited for 800 mapped reads to optimize the visualizations. **Figure S6.** Identification of protein-coding genes located in B chromosomes of the *A. correntinus*, using the number of mapped reads that map to the CDSs found in the transcriptome, in the 0B (X axis) and 1B (Y axis). Each dot represents a coding sequence with only those labeled that recorded the log2 greater than 1. The plot is limited for 800 mapped reads to optimize the visualizations. **Figure S7.** Coverage plots of (representative) coding sequences detected on the B chromosome of *A. mexicanus* using Log base 2 ratio. Each plot compares the reads depth of the transcript between 0B and 2B. **Figure S8**. Coverage plots of (representative) coding sequences detected on the B chromosome of *A. correntinus* using Log base 2 ratio. Each plot compares the reads depth of the transcript between 0B and 1B. **Figure S9**. Coverage plots of (representative) coding sequences detected on the B chromosome of *A. flavolineata* using Log base 2 ratio. Each plot compares the reads depth of the transcripts between 0B and 2B. **Figure S10.** Double FISH mapping of candidate blocks in *A. flavolineata*. Each panel represents mapping of two distinct blocks (7 and 14 – See Supplementary Table S2) labeled with digoxigenin (block 7 in red) and biotin (block 14 in green). A scattered pattern of markings of block 7 can be observed for the B chromosomes (white arrows), whereas telomeric and centromeric associated signals of blocks 7 and 14 are obvious for different A and X chromosomes. **Figure S11**. The methylation profile of microB blocks in *A. mexicanus*. (**A**) Graph shows the number of Bisulphite reads analyzed with B blocks and alignments. (**B**) Pie chart represents percentage of B block methylated Cs in different contexts with the highest percent in CpGs regions. (**C**) Pie chart highlights the number of hypomethylated (less than 50% methylated Cs), unmethylated, highly methylated and hypermethylated (more than 90% methylated Cs) B chromosome blocks. (**D**) Bisuphite coverage plots of hypermethylated blocks are shown as examples. Refer to Supplementary datasets (excel) for a complete list of methylated blocks with methylation level. **Figure S12**. FISH mapping of 45S rDNA (red labeling) in *A. mexicanus*. The micro B is indicated with an arrow showing no sign of 45S rDNA. **Figure S13.** Synteny dotplot of whole genome aligments between de novo B+ assembly and reference masked (coding) genome of *A. mexicanus*. **Figure S14.** Violin plots show the distribution of length for various types of genomic rearrangements from 0 to 5000 bp range. **Figure S15.** Synteny dotplot (synmap version, unfiltered) of self alignments of B blocks (*A. mexicanus*). **Figure S16.** Synteny dotplot (synmap version, unfiltered) of self alignments of B blocks (*A. correntinus*). **Figure S17.** Synteny dotplot (synmap version, unfiltered) of self alignments of B blocks (*A. flavolineata*).

**Additional file 4: Supplementary Datasets** (Excel files may include multiple sheets for respective species). **Dataset 1.** A complete list of B blocks detected in *A. mexicanus*, *A. correntinus* and *A. flavolineata*. **Dataset 2**. Genes integrity percentage of the B blocks of *A. mexicanus* and *A. correntinus*. **Dataset 3.** A complete list of B-linked CDS with significant Log base 2 ratio in *A. mexicanus*, A correntinus and *A. flavolineata*. **Dataset 4.** List of B exclusive sequences detected in *A. mexicanus*, *A. correntinus* and *A. flavolineata*. **Dataset 5.** qPCR GDR ratio experiment CT values of representative B blocks in 0B, 1B and 2B of *A. mexicanus*. **Dataset 6.** Methylated B blocks and CpG Islands detected in *A. mexicanus*. **Dataset 7.** A complete list of enriched GO terms in all studied species. **Dataset 8**. Fasta sequences of the B blocks (*A. mexicanus*). **Dataset 9**. Fasta sequences of the B blocks (*A. correntinus*). **Dataset 10.** Fasta sequences of the B blocks (*A. flavolineata*). **Dataset 11.** Fasta sequences of B exclusive sequences (*A. mexicanus*). **Dataset 12.** Fasta sequences of B exclusive sequences (*A. correntinus*). **Dataset 13.** Fasta sequences of B exclusive sequences (*A. flavolineata*).

## Data Availability

The complete sequencing data and genome assembly generated in the present study has been publicly made available at NCBI Bioproject (PRJNA606808). The sequencing reads of the B- and B+ samples of *A. mexicanus*, *A. correntinus* and *A. flavolineata* can be accessed with accession IDs as: SRR11095917, SRR11095918, SRR11095919, SRR11147339, SRR11147340, SRR11148657, SRR11148656, SRR11148655 respectively. The *de novo* genome assembles can be accessed by SAMN14118361 and SAMN14120472. (Data has been submitted in NCBI and will be released public immediately after publication of this study). Custom scripts and commands are available at github (https://github.com/farhan-phd/Integrative-genomic-analysis-reveals-the-gene-contents-repeats-landscapes-and-evolutionary-dynamics/tree/master).

## Abbreviations

B-blocks: B chromosome blocks
bp: Base pair
BPR: B+ ratio
CDS: Coding sequences
DAPI: 4′,6-diamidino-2 phenylindole
FISH: Fluorescence in situ hybridization
GO: Gene ontologies
kb: Kilo bases
NC: Normalized coverage
NGS: Next generation sequencing
MC: Mean coverage
MRSD: Standard deviation
qPCR: Quantitative Real-Time PCR

## References

[1] Longley AE (1927). Supernumerary chromosomes in *Zea mays*. J Agric Res.

[2] Ahmad S, Martins C (2019). The modern view of B chromosomes under the impact of high scale omics analyses. Cells.

[3] Wilson EB (1909). Studies on chromosomes. V. the chromosomes of *metapodius*. A contribution to the hypothesis of the genetic continuity of chromosomes. J Exp Zool.

[4] Camacho JPM, Gregory TR (2005). B chromosomes. The evolution of the genome.

[5] D’Ambrosio U, Alonso-Lifante MP, Barros K, Kovařík A, Mas de Xaxars G, Garcia S (2017). B-chrom: A database on B-chromosomes of plants, animals and fungi. New Phytol.

[6] Jones RN, Rees H. B chromosomes. London: Academic; 1982.

[7] Randolph LF. Genetic characteristics of the B chromosomes in maize. Genetics. 1941;26:608–31. 10.1093/genetics/26.6.608 PMC1224308 17247025

[8] Nur U, Werren JH, Eickbush DG, Burke WD, Eickbush TH (1988). A “selfish” B chromosome that enhances its transmission by eliminating the paternal genome. Science.

[9] Leach CR, Houben A, Field B, Pistrick K, Demidov D, Timmis JN (2005). Molecular evidence for transcription of genes on a B chromosome in *Crepis capillaris*. Genetics.

[10] Graphodatsky AS, Kukekova AV, Yudkin DV, Trifonov VA, Vorobieva NV, Beklemisheva VR (2005). The proto-oncogene C-KIT maps to canid B-chromosomes. Chromosom Res.

[11] Ruiz-Estévez M, López-León MD, Cabrero J, Camacho JP (2012). B-chromosome ribosomal DNA is functional in the grasshopper *Eyprepocnemis plorans*. PLoS One.

[12] Trifonov VA, Dementyeva PV, Larkin DM, O’Brien PCM, Perelman PL, Yang F (2013). Transcription of a protein-coding gene on B chromosomes of the Siberian roe deer (*Capreolus pygargus*). BMC Biol.

[13] Mestriner CA, Galetti PM, Valentini SR, Ruiz IRG, Abel LDS, Moreira-Filho O (2000). Structural and functional evidence that a B chromosome in the characid fish *Astyanax scabripinnis* is an isochromosome. Heredity.

[14] Silva DMZA, Utsunomia R, Pansonato-Alves JC, Oliveira C, Foresti F (2015). Chromosomal mapping of repetitive DNA sequences in five species of *Astyanax* (Characiformes, Characidae) reveals independent location of U1 and U2 snRNA sites and association of U1 snRNA and 5S rDNA. Cytogenet Genome Res.

[15] Duílio DMZ, Daniel SN, Camacho JPM, Utsunomia R, Ruiz-Ruano FJ, Penitente M (2016). Origin of B chromosomes in the genus *Astyanax* (Characiformes, Characidae) and the limits of chromosome painting. Mol Gen Genomics.

[16] Pazza R, Dergam JA, Kavalco KF. Trends in karyotype evolution in *Astyanax* (Teleostei, Characiformes, Characidae): insights from molecular data. Front Genet. 2018;9:131. 10.3389/fgene.2018.00131 PMC5911472 29713335

[17] Paiz LM, Baumgärtner L, da Graça WJ, Margarido VP (2015). Basic cytogenetics and physical mapping of ribosomal genes in four *Astyanax* species (Characiformes, Characidae) collected in middle Paraná river, Iguassu National Park: considerations on taxonomy and systematics of the genus. Comp Cytogenet.

[18] Romero A, Paulson KM (2001). It’s a wonderful hypogean life: a guide to the troglomorphic fishes of the world. Environ Biol Fish.

[19] Jeffery WR (2001). Cavefish as a model system in evolutionary developmental biology. Dev Biol.

[20] Kavalco KF, De Almeida-Toledo LF (2007). Molecular cytogenetics of blind Mexican tetra and comments on the karyotypic characteristics of genus *Astyanax* (Teleostei, Characidae). Zebrafish.

[21] De Silva DMZA, Utsunomia R, Ruiz-Ruano FJ, Daniel SN, Porto-Foresti F, Hashimoto DT, et al. High-throughput analysis unveils a highly shared satellite DNA library among three species of fish genus *Astyanax*. Sci Rep. 2017;7:12726. 10.1038/s41598-017-12939-7 PMC5635008 29018237

[22] Palestis BG, Cabrero J, Trivers R (2010). Prevalence of B chromosomes in Orthoptera is associated with shape and number of a chromosomes. Genetica.

[23] Cella DM, Ferreira A (1991). The cytogenetics of *Abracris flavolineata* (Orthoptera, Caelifera, Ommatolampinae, Abracrini). Rev Bras Genet.

[24] Bueno D, Palacios-Gimenez OM, Cabral-de-Mello DC. Chromosomal mapping of repetitive DNAs in the grasshopper *Abracris flavolineata* reveal possible ancestry of the B chromosome and H3 histone spreading. PLoS One. 2013;8(6):e66532. 10.1371/journal.pone.0066532 PMC3694960 23826099

[25] Milani D, Bardella VB, Ferretti ABSM, Palacios-Gimenez OM, Melo ADS, Moura RC, Loreto V, Song H, Cabral-de-Mello DC (2018). Satellite DNAs unveil clues about the ancestry and composition of B chromosomes in three grasshopper species. Genes.

[26] Ruiz-Ruano FJ, Castillo-Martínez J, Cabrero J, Gómez R, Camacho JPM, López-León MD (2018). High-throughput analysis of satellite DNA in the grasshopper *Pyrgomorpha conica* reveals abundance of homologous and heterologous higher-order repeats. Chromosoma.

[27] Potapov VA, Solovyev VV, Romanshchenko AG, Sosnovtsev SV, Ivanov SV (1991). The structural and evolutional features of complex tandemly arranged Bsp-repeats in fox genome. II. Tissue-specific and recombinational sites in the BamHI-dimer. Mol Biol.

[28] Peppers JA, Wiggins LE, Baker RJ (1997). Nature of B chromosomes in the harvest mouse *Reithrodontomys megalotis* by fluorescence in situ hybridization (FISH). Chromosom Res.

[29] Wurster-Hill DH, Ward OG, Davis BH, Park JP, Moyzis RK, Meyne J (1988). Fragile sites, telomeric DNA sequences, B chromosomes, and DNA content in raccoon dogs, *Nyctereutes procyonoides*, with comparative notes on foxes, coyote, wolf, and raccoon: (with i color plate). Cytogenet Genome Res.

[30] Szczerbal I, Switonski M (2003). B chromosomes of the Chinese raccoon dog (*Nyctereutes procyonoides procyonoides* Gray) contain inactive NOR-like sequences. Caryologia.

[31] Stitou S (2000). Díaz De La Guardia R, Jiménez R, Burgos M. inactive ribosomal cistrons are spread throughout the B chromosomes of *Rattus rattus* (Rodentia, Muridae). Implications for their origin and evolution. Chromosom Res.

[32] Rubtsov NB, Karamysheva TV, Andreenkova OV, Bochkaerev MN, Kartavtseva IV, Roslik GV (2004). Comparative analysis of micro and macro B chromosomes in the Korean field mouse *Apodemus peninsulae* (Rodentia, Murinae) performed by chromosome microdissection and FISH. Cytogenet Genome Res.

[33] Teruel M, Cabrero J, Perfectti F, Camacho JPM (2010). B chromosome ancestry revealed by histone genes in the migratory locust. Chromosoma.

[34] Trifonov VA, Perelman PL, Kawada SI, Iwasa MA, Oda SI, Graphodatsky AS (2002). Complex structure of B-chromosomes in two mamalian species: *Apodemus peninsulae* (Rodentia) and *Nyctereutes procyonoides* (Carnivora). Chromosom Res.

[35] Martis MM, Klemme S, Banaei-Moghaddam AM, Blattner FR, Macas J, Schmutzer T (2012). Selfish supernumerary chromosome reveals its origin as a mosaic of host genome and organellar sequences. Proc Natl Acad Sci U S A.

[36] Valente GT, Conte MA, Fantinatti BEA, Cabral-De-Mello DC, Carvalho RF, Vicari MR (2014). Origin and evolution of B chromosomes in the cichlid fish Astatotilapia latifasciata based on integrated genomic analyses. Mol Biol Evol.

[37] Navarro-Domínguez B, Ruiz-Ruano FJ, Cabrero J, Corral JM, López-León MD, Sharbel TF, et al. Protein-coding genes in B chromosomes of the grasshopper Eyprepocnemis plorans. Sci Rep. 2017;7:45200. 10.1038/srep45200 PMC5377258 28367986

[38] Huang W, Du Y, Zhao X, Jin W. B chromosome contains active genes and impacts the transcription of A chromosomes in maize (*Zea mays* L.). BMC Plant Biol. 2016;16:88. 10.1186/s12870-016-0775-7 PMC4833949 27083560

[39] Li Y, Jing XA, Aldrich JC, Clifford C, Chen J, Akbari OS (2017). Unique sequence organization and small RNA expression of a “selfish” B chromosome. Chromosoma.

[40] Aldrich JC, Leibholz A, Cheema MS, Ausió J, Ferree PM. A “selfish” B chromosome induces genome elimination by disrupting the histone code in the jewel wasp *Nasonia vitripennis*. Sci Rep. 2017;7:42551. 10.1038/srep42551 PMC5304203 28211924

[41] Ramos É, Cardoso AL, Brown J, Marques DF, Fantinatti BEA, Cabral-de-Mello DC (2017). The repetitive DNA element BncDNA, enriched in the B chromosome of the cichlid fish *Astatotilapia latifasciata*, transcribes a potentially noncoding RNA. Chromosoma.

[42] Navarro-Domínguez B, Martín-Peciña M, Ruiz-Ruano FJ, Cabrero J, Corral JM, López-León MD (2019). Gene expression changes elicited by a parasitic B chromosome in the grasshopper *Eyprepocnemis plorans* are consistent with its phenotypic effects. Chromosoma.

[43] Hong ZJ, Xiao JX, Peng SF, Lin YP, Cheng YM (2020). Novel B-chromosome-specific transcriptionally active sequences are present throughout the maize B chromosome. Mol Gen Genomics.

[44] Novák P, Neumann P, Pech J, Steinhaisl J, MacAs J (2013). RepeatExplorer: a galaxy-based web server for genome-wide characterization of eukaryotic repetitive elements from next-generation sequence reads. Bioinformatics.

[45] Gore AV, Tomins KA, Iben J, Ma L, Castranova D, Davis AE (2018). An epigenetic mechanism for cavefish eye degeneration. Nat Ecol Evol.

[46] Goel M, Sun H, Jiao W (2019). SyRI: finding genomic rearrangements and local sequence differences from whole-genome assemblies. Genome Biol.

[47] McGaugh SE, Gross JB, Aken B, Blin M, Borowsky R, Chalopin D, et al. The cavefish genome reveals candidate genes for eye loss. Nat Commun. 2014;5:5307. 10.1038/ncomms6307 PMC4218959 25329095

[48] Venkatesh B, Kirkness EF, Loh YH, Halpern AL, Lee AP, Johnson J (2007). Survey sequencing and comparative analysis of the elephant shark (Callorhinchus milii) genome. PLoS Biol.

[49] Star B, Nederbragt AJ, Jentoft S, Grimholt U, Malmstrøm M, Gregers TF (2011). The genome sequence of Atlantic cod reveals a unique immune system. Nature.

[50] Jones FC, Grabherr MG, Chan YF, Russell P, Mauceli E, Johnson J (2012). The genomic basis of adaptive evolution in threespine sticklebacks. Nature.

[51] Howe K, Clark MD, Torroja CF, Torrance J, Berthelot C, Muffato M (2013). The zebrafish reference genome sequence and its relationship to the human genome. Nature.

[52] Smith JJ, Kuraku S, Holt C, Sauka-Spengler T, Jiang N, Campbell MS (2013). Sequencing of the sea lamprey (Petromyzon marinus) genome provides insights into vertebrate evolution. Nat Genet.

[53] Friebe B, Jiang J, Gill B (1995). Detection of 5S rDNA and other repeated DNA on supernumerary B chromosomes of Triticum species (Poaceae). Plant Syst Evol.

[54] Cabrero J, Bakkali M, Bugrov A, Warchalowska-Sliwa E, López-León MD, Perfectti F (2003). Multiregional origin of B chromosomes in the grasshopper Eyprepocnemis plorans. Chromosoma.

[55] Bugrov AG, Karamysheva TV, Perepelov EA, Elisaphenko EA, Rubtsov DN, Warchałowska-Śliwa E (2007). DNA content of the B chromosomes in grasshopper *Podisma kanoi* Storozh. (Orthoptera, Acrididae). Chromosom Res.

[56] Oliveira NL, Cabral-De-Mello DC, Rocha MF, Loreto V, Martins C, Moura RC. Chromosomal mapping of rDNAs and H3 histone sequences in the grasshopper *Rhammatocerus brasiliensis* (acrididae, gomphocerinae): extensive chromosomal dispersion and co-localization of 5S rDNA/H3 histone clusters in the a complement and B chromosome. Mol Cytogenet. 2011;4:24. 10.1186/1755-8166-4-24 PMC3234176 22075079

[57] Kour G, Kaul S, Dhar MK (2014). Molecular characterization of repetitive DNA sequences from B chromosome in *Plantago lagopus* L. Cytogenet Genome Res.

[58] Houben A, Banaei-Moghaddam AM, Klemme S, Timmis JN. Evolution and biology of supernumerary B chromosomes. Cell Mol Life Sci. 2014;71(3):467–78. 10.1007/s00018-013-1437-7 PMC11113615 23912901

[59] Coan RLB, Martins C (2018). Landscape of transposable elements focusing on the B chromosome of the cichlid fish *Astatotilapia latifasciata*. Genes.

[60] Banaei-Moghaddam AM, Meier K, Karimi-Ashtiyani R, Houben A (2013). Formation and expression of pseudogenes on the B chromosome of rye. Plant Cell.

[61] Yoshida K, Terai Y, Mizoiri S, Aibara M, Nishihara H, Watanabe M (2011). B chromosomes have a functional effect on female sex determination in Lake Victoria cichlid fishes. PLoS Genet.

[62] Jehangir M, Ahmad SF, Cardoso AL, Ramos E, Valente GT, Martins C (2019). De novo genome assembly of the cichlid fish *Astatotilapia latifasciata* reveals a higher level of genomic polymorphism and genes related to B chromosomes. Chromosoma.

[63] Protas M, Conrad M, Gross JB, Tabin C, Borowsky R (2007). Regressive evolution in the Mexican cave tetra, *Astyanax mexicanus*. Curr Biol.

[64] Castro JP, Hattori RS, Yoshinaga TT, de A Silva DMZ, Ruiz-Ruano FJ, Foresti F, et al. Differential expression of genes related to sexual determination can modify the reproductive cycle of *Astyanax scabripinnis* (Characiformes: Characidae) in b chromosome carrier individuals. Genes (Basel). 2019;10. 10.3390/genes10110909 PMC6896079 31717315

[65] Clemente M, Garma C, De Sola BG, Henriques-Gil N (2001). Steep variation in mitochrondrial DNA and B chromosomes among natural populations of *Eyprepocnemis plorans* (Acrididae). Hereditas.

[66] Ma W, Gabriel TS, Martis MM, Gursinsky T, Schubert V, Vrána J (2017). Rye B chromosomes encode a functional Argonaute-like protein with in vitro slicer activities similar to its a chromosome paralog. New Phytol.

[67] Sumner AT (1972). A simple technique for demonstrating centromeric heterochromatin. Exp Cell Res.

[68] Gordon A, Hannon GJ (2014). FASTX-Toolkit.

[69] Bolger AM, Lohse M, Usadel B (2014). Trimmomatic: a flexible trimmer for Illumina sequence data. Bioinformatics.

[70] Andrews S (2010). FastQC: a quality control tool for high throughput sequence data.

[71] Cunningham F, Achuthan P, Akanni W, Allen J, Amode MR, Armean IM (2019). Ensembl 2019. Nucleic Acids Res.

[72] Luo R, Liu B, Xie Y, Li Z, Huang W, Yuan J (2012). SOAPdenovo2: an empirically improved memory-efficient short-read de novo assembler. Gigascience.

[73] Langmead B, Salzberg SL (2012). Fast gapped-read alignment with bowtie 2. Nat Methods.

[74] Alonge M, Soyk S, Ramakrishnan S, Wang X, Goodwin S, Sedlazeck FJ (2019). RaGOO: fast and accurate reference-guided scaffolding of draft genomes. Genome Biol.

[75] Gurevich A, Saveliev V, Vyahhi N, Tesler G (2013). QUAST: quality assessment tool for genome assemblies. Bioinformatics.

[76] Quinlan AR, Hall IM (2010). BEDTools: a flexible suite of utilities for comparing genomic features. Bioinformatics.

[77] Skinner ME, Uzilov AV, Stein LD, Mungall CJ, Holmes IH (2009). JBrowse: a next-generation genome browser. Genome Res.

[78] Phanstiel DH, Boyle AP, Araya CL, Snyder MP (2014). Sushi.R: flexible, quantitative and integrative genomic visualizations for publication-quality multi-panel figures. Bioinformatics.

[79] Smit A, Hubley R, Green P (2013). RepeatMasker Open-4.0. 2013–2015.

[80] Pinkel D, Straume T, Gray JW (1986). Cytogenetic analysis using quantitative, high-sensitivity, fluorescence hybridization. Proc Natl Acad Sci U S A.

[81] Nguyen P, Sykorova M, Sichova J, Kůta V, Dalíková M, Čapková Frydrychová R, Neven LG, Sahara K, Marec F (2013). Neo-sex chromosomes and adaptive potential in tortricid pests. Proc Natl Acad Sci U S A.

[82] Montiel EE, Cabrero J, Ruiz-Estévez M, Burke WD, Eickbush TH, Camacho JPM (2014). Preferential occupancy of R2 retroelements on the B chromosomes of the grasshopper *Eyprepocnemis plorans*. PLoS One.

[83] Vij S, Kuhl H, Kuznetsova IS, Komissarov A, Yurchenko AA, Van Heusden P (2016). Correction: chromosomal-level assembly of the Asian Seabass genome using long sequence reads and multi-layered scaffolding. PLoS Genet.

[84] Rajičić M, Romanenko SA, Karamysheva TV, Blagojević J, Adnadević T, Budinski I, et al. The origin of B chromosomes in yellownecked mice (*Apodemus flavicollis*)-break rules but keep playing the game. PLoS One. 2017;12(3):e0172704. 10.1371/journal.pone.0172704 PMC5362141 28329013

[85] Martin M (2011). Cutadapt removes adapter sequences from high-throughput sequencing reads. EMBnet.journal.

[86] Wang X, Fang X, Yang P, Jiang X, Jiang F, Zhao D (2014). The locust genome provides insight into swarm formation and long-distance flight. Nat Commun.

[87] Church DM, Schneider VA, Graves T, Auger K, Cunningham F, Bouk N (2011). Modernizing reference genome assemblies. PLoS Biol.

[88] Huang X, Madan A (1999). CAP3: a DNA sequence assembly program. Genome Res.

[89] Altschul SF, Gish W, Miller W, Myers EW, Lipman DJ (1990). Basic local alignment search tool. J Mol Biol.

[90] Brionne A, Juanchich A, Hennequet-Antier C. ViSEAGO: a bioconductor package for clustering biological functions using gene ontology and semantic similarity. BioData Min. 2019;12:16. 10.1186/s13040-019-0204-1 PMC6685253 31406507

[91] McDonald J. Fisher’s exact test of independence. Handbook of biological statistics. Baltimore: Sparky House Publ; 2014.

[92] Wang JZ, Du Z, Payattakool R, Yu PS, Chen CF (2007). A new method to measure the semantic similarity of GO terms. Bioinformatics.

[93] Krueger F, Andrews SR (2011). Bismark: a flexible aligner and methylation caller for bisulfite-Seq applications. Bioinformatics.

[94] Kurtz S, Phillippy A, Delcher AL, Smoot M, Shumway M, Antonescu C, et al. Versatile and open software for comparing large genomes. Genome Biol. 2004;5:R12. 10.1186/gb-2004-5-2-r12 PMC395750 14759262

[95] Cheong WH, Tan YC, Yap SJ, Ng KP (2015). ClicO FS: An interactive web-based service of Circos. Bioinformatics.

[96] Haug-Baltzell A, Stephens SA, Davey S, Scheidegger CE, Lyons E (2017). SynMap2 and SynMap3D: web-based whole-genome synteny browsers. Bioinformatics.

[97] Lysak MA, Berr A, Pecinka A, Schmidt R, McBreen K, Schubert I (2006). Mechanisms of chromosome number reduction in *Arabidopsis thaliana* and related *Brassicaceae* species. Proc Natl Acad Sci U S A.

