# Supplementary material for: B chromosomes of multiple species have intense evolutionary dynamics and accumulated genes related to important biological processes
Source: BMC Genomics. 2020 Sep 23;21:656. doi: 10.1186/s12864-020-07072-1 (PMC7509943; doi:10.1186/s12864-020-07072-1)
Supplement: Supplementary file 1 — Additional file 1. Supplementary Note 1. [file 12864_2020_7072_MOESM1_ESM.pdf]

Supplementary Note 1

The *de novo* assemblies of *A. mexicanus* contain 1.4 and 1.6 Gb sequences anchored in 25 chromosomes (linkage groups) with 1,854 and 1,920 unplaced scaffolds for both B- and B+ genomes respectively. While that of *A. correntinus* have 25 linkage groups with 1,854 and 1,900 unplaced scaffolds for both B- and B+ genomes respectively. Assembly assessment of these genomes confirmed a high level of N50 (Supplementary Table S1), thus indicating the completeness of these assemblies. In this way, a total four *de novo* highly continuous reference guided de novo assemblies were generated.

To generate the draft version genome of *A. flavolineata* we merged the Illumina reads of 0B (19.9×), 1B (10.79×) and 2B (10.26×) reads to achieve sufficient reads coverage (total 40.95×) for the de novo assembly. We used kmer of 63 for this assembly as determined by kmergenie tool. The total genome spans around 6.3 Gb, however due to the fragmented assembly the genome size could be underrepresented. The genome evaluation estimated a lower N50 value (3.3 kb) indicating a high level of misassemblies and broken scaffolds.
