# Supplementary material for: B chromosomes of multiple species have intense evolutionary dynamics and accumulated genes related to important biological processes
Source: BMC Genomics. 2020 Sep 23;21:656. doi: 10.1186/s12864-020-07072-1 (PMC7509943; doi:10.1186/s12864-020-07072-1)
Supplement: Supplementary file 2 — Additional file 2: Table S1. De novo genome assemblies and their statistics. Table S2. List of primers of representative blocks used in qPCR and FISH mapping. Table S3. Summary of analyzed data used for microdissected Bs assemblies. Table S4. List of 10 common functions shared among the Bs of all seven analyzed species. [file 12864_2020_7072_MOESM2_ESM.pdf]

Supplementary Tables

Table S1. De novo genome assemblies and their statistics

**Organism**	**LGs/Chr**	**Unplaced scaffolds**	**N50**	**N75**	**GC%**	**Genome size**
A. mexicanus (B-)	25	1854	36.3 Mb	24.4 Mb	37.90	1.4 Gb
A. mexicanus (B+)	25	1920	39.6 Mb	26.13 Mb	37.93	1.6 Gb
A. correntinus (B-)	18	1861	1617 Mb	27.3 Mb	37.70	2.3 Gb
A. correntinus (B+)	18	1907	1916 Mb	28.1 Mb	37.78	2.6 Gb
A. flavolineata (B+)	N/A	1,727,319 (greater than 1kb)	3.3 kb	1.5 kb	41.06	6.3 Gb

Table S2. List of primers of representative blocks used in qPCR and FISH mapping

Species	Block Name	Block ID	Forward primer	Reverse Primer	Product size (bp)	Melting temperature	Primers type
A . mexicanus	NC_035906.1_10647615-10648143	Block-1-FISH	ACATTTCCTGCCTTTTAGTTGGG	ATGACCAATTCATAGACAAGTGTCAC	336	59.3 (F); 59.6 (R)	FISH
	NC 035906.1 10647615-10648143	Block-1-qPCR	ACATTTCCTGCCTTTTAGTTGGG	TCATGTCACGTACACTTCACAGT	151	59.4 (F); 60 (R)	qPCR
	NC_035913.1_22862787-22865602	Block-2-FISH	CCATCAGTCAGCATCCTGTTTC	GTGGCACCACTTTAAAATAAGGCT	267	59.1 (F); 59.8 (R)	FISH
	NC_035913.1_22862787-22865602	Block-2-qPCR	CCATCAGTCAGCATCCTGTTTC	GCGACTAAGCACTTCATAAACACA	164	59.32 (F); 59.8 (R)	qPCR
	NC_035913.1_22871651-22872613	Block-3-FISH	CACACCTATTCACTCCCTTGGT	GCAGTACACAAGCAAATGACAAGA	329	59.7 (F); 60.3 (R)	FISH
	NC 035913.1 22871651-22872613	Block-3-qPCR	CACACCTATTCACTCCCTTGGT	gtCCTAAGCGTGGTTAAAGTATTACC	152	59.7 (F); 59.7 (R)	qPCR
	NC_035913.1_22873870-22874705	Block-4-FISH	AGTTGTTACCTGTTTACCCAACATTAG	ACGAATCATGCACTCTCACCA	406	59.8 (F); 59.7 (R)	FISH
	NC_035913.1_22873870-22874705	Block-4-qPCR	AGTTGTTACCTGTTTACCCAACATTAG	ACATGAGGTAGTACATTAACAACGC	180	59.8 (F); 59.4 (R)	qPCR
	NC_035913.1_23188499-23195606	Block-5-FISH	GGAGGGTAGATGGGGAAGGA	TCAGTTAGATAGAAGCTGGATGTTGT	249	59.7 (F); 59.8 (f)	FISH
	NC_035913.1_23188499-23195606	Block-5-qPCR	GGAGGGTAGATGGGGAAGGA	AAACCCTGGTCTCTGCCCTA	180	59.73 (F); 60.18 (R)	qPCR
	NW_019170575.1-27717_28646	Block-6-FISH	AAGAGGCGATTCTGCGGAA	CGCCTCCTCAAGAACAGCAA	536	60.0 (F); 60.6 (R)	FISH
	NW_019170575.1-27717_28646	Block-6-qPCR	AAGAGGCGATTCTGCGGAA	ACCCTCGGATGTGGTGATTC	205	60.0 (F); 59.46 (R)	qPCR
	NW 019170910.1 26677-27858	Block-7-FISH	ACAGTTCCACCTCAGTCTCAC	CTTGGGTTCTGTAGCAGGACA	537	59.3 (F); 59.7 (R)	FISH
	NW_019170910.1_26677-27858	Block-7-qPCR	ACAGTTCCACCTCAGTCTCAC	AGACACAGCAGAGCTACGGT	157	59.3 (F); 60.9 (R)	qPCR
	NW_019171087.1_32674-33258	Block-8-qPCR/FISH	GGGAATGCTCACAGGCTGA	ACACGGGTTTGAAAAATACCAACT	103	59.70 (F); 59.60 (R)	qPCR/FISH
	NW_019171959.1_9110-10065	Block-9-FISH	ATCGCGGGCAAATGTCCAAT	GACATAGGTGATACCCGGAAACA	323	61.0 (F); 60 (R)	FISH
	NW_019171959.1_9110-10065	Block-9-qPCR	ATCGCGGGCAAATGTCCAAT	GGGTCACAGCAGTCATACGG	183	61.0 (F); 60.5 (R)	qPCR
	NW_019172828.1_4736897-4738479	Block-10-FISH	GCGGACATTTCCTGCCTTTT	TCCATAGTGAAACGGCGTGA	617	59.40 (F); 59.40 (R)	FISH
	NW_019172828.1_4736897-4738479	Block-10-qPCR	GCGGACATTTCCTGCCTTTT	TGACAGCACAGAGACAATCATCA	117	59.40 (F); 60.0 (F)	qPCR
	NW_019172877.1_4865-5684	Block-11-FISH	gACTTTCAATCCCATGTTTGCCA	CCCTGAATACCCAGTTTTTCCC	493	60 (F); 60.5 (R)	FISH
	NW_019172877.1_4865-5684	Block-11-qPCR	gACTTTCAATCCCATGTTTGCCA	aGCACCCCATTGCATCATGG	173	60 (F); 61 (R)	qPCR
A. correntinus	Gypsy	33-I_DR 498 903	ACGGACTGGGCTTTCCATTT	CGTCCGGAACACAAAACGAA	406	59.3 (F) 59.6 (R)	FISH
	Tc-Mariner	DR 723 1024	GCTGCTCGAGGTCTAGTGTG	TTGGTACTTTTGGCAGTGTGG	252	59.4 (F) 60 (R)	FISH

A. flavolineata	Scaffold2635:1157-2411	Block 1	TCGCATAGGGAATTGTGGGT	CTTCTCTACGCCTGGGACAC	507	59.08<br>59.83	FISH
	Scaffold122200:629-1783	Block 2	CGCCATTGTTTGTCCGACTC	ATGGGTGGACTTTGTTCGCT	539	59.83<br>59.89	FISH
	Scaffold171457:539-1028	Block 3	GCTCCAAATTTTCGTCATTCTTCG	TTCGCATTCGGTCTCGATGT	401	59.45<br>59.83	FISH
	Scaffold259420:93-1278	Block 4	ATCTGTGCCTTGACGACAGG	AAACACGGCTCTGCTACTGT	561	60.04<br>59.61	FISH
	Scaffold259420:2889-3469	Block 5	CCACCAAGATCACCAGACCTA	GAGGGGTTGTTCAAACACAGC	522	58.81<br>59.93	FISH
	Scaffold399583:53-2099	Block 6	TCCCTGGAGTCATGGACTGT	GGGATTGTCACCCTTCGTGT	510	59.88<br>59.96	FISH
	Scaffold399583:5970-8845	Block 7	GAGATATCGGCTGGCGACAA	TTCGCCGTTAATGCCAGACT	505	59.97<br>60.04	FISH
	Scaffold401546:52-762	Block 8	ACGGGAAGATGGCAGATAGC	TCCACCTCATCAACTTTCCAA	586	59.61<br>57.13	FISH
	Scaffold623432:4237-5208	Block 9	AGAAGAGTCCCTGCACAACG	GCAGACGACCCTCATCAACA	553	59.97<br>60.04	FISH
	Scaffold723254:13-660	Block 10	CGCCCGTTTTCATGCTTGT	GGAGCAAACAACTTACACG	426	59.71<br>59.5	FISH
	Scaffold767652:3050-3880	Block 11	AGATTGTGACGCCAAGCTCA	ACGCCTAGCACCTGGAAATC	558	59.96<br>60.11	FISH
	Scaffold848713:4910-8047	Block 12	GCTCGATACGAAGCACCTGA	GCCTCCACGTCTATGAACCC	540	59.90<br>60.18	FISH
	Scaffold1438633:3-1038	Block 13	ATGCAGACTGGGTCTCAACG	CTTGACACAGCCGCTCAAAC	602	60.04<br>60.04	FISH
	Scaffold1632105:2434-3149	Block 14	GGGTGAATCCTGGTTAGACTCT	CGAGAGGTGTGCTAAACAATGG	528	58.89<br>59.58	FISH

Table S3. Summary of analyzed data used for microdissected Bs assemblies.

**Microdissected B/species/ Acession ID:**	**Raw reads/Decontaminated trimmed reads**	**Assembly**	**Spacer length**	**Fragments**	**Total length (bp)**	**% of reference genome**
B1/ Asian Seabass fish/ Acession ID: SRX2041358 Species: *L. calcarifer*	876,260/78,611	ChB1 pseudo-scaffolds	10 bp	1,764	59,065	0.010
			100 bp	1,743	603,58	0.010
			2 kb	1,662	963,69	0.016
			10 kb*	1,412	434,598	0.074
			100 kb	1213	21,578,827	3.67
B2/B3 Asian Seabass fish/ Acession ID: SRX2041352/SRX1484569 Species: *L. calcarifer*	765,254/65,780	ChB2-B3 pseudo-scaffolds	10 bp	1,552	71,373	0.012
			100 bp	1530	72,775	0.012
			2 kb	1412	164,958	0.028
			10 kb*	1,601	378,367	0.064
			100 kb	1,097	16,912,139	2.9
B4/ Grasshopper/ Acession ID: SRX3412298 Species: *E. plorans*	69,279,084/67,469,719	ChB4 pseudo-scaffolds	10 bp	5,006,153	5,188,127	0.08
			100 bp	4,586,192	7,471,409	0.11
			2 kb	1,460,148	23,634,021	2.3
			10 kb*	969,779	25,972,513	2.5
			100 kb	725,481	383,082,448	38.3
B5/ Mouse/Acession ID: SRX3412297 Species: *Apodemus peninsulae*	938,990/660,346	ChB5 pseudo-scaffolds	10 bp	94	14,095	-
			100 bp	93	14,127	-

			2 kb	84	19,715	-
			10 kb*	82	29,798	-
			100 kb	64	800,520	-
B6/ Mouse/Acession ID: SRX3412293	2630300/113844	ChB5 pseudo-scaffolds	10 bp	40	6,183	-
Species: *Apodemus peninsulae*			100 bp	40	6,183	-
			2 kb	40	6,183	-
			10 kb*	39	10,445	-
			100 kb	36	132,972	-
B7/ Mouse/Acession ID:SRX3412299<br>Species: *A. flavicollis*	1590728/783058	ChB7 pseudo-scaffolds	10 bp	901	70,503	-
			100 bp	889	71,210	-
			2 kb	854	92,874	-
			10 kb*	773	457,733	-
			100 kb	375	18,833,514	-

10 kb* was considered for annotation and gene ontologies

Table S4. List of 10 common functions shared among the Bs of all seven analyzed species

GO IDs	GO term	Definition
GO:0016740	Transferase activity	Catalysis of the transfer of a group, e.g. a methyl group, glycosyl group, acyl group, phosphorus-containing, or other groups, from one compound (generally regarded as the donor) to another compound (generally regarded as the acceptor). Transferase is the systematic name for any enzyme of EC class 2.
GO:0016021	Integral component of membrane	The component of a membrane consisting of the gene products and protein complexes having at least some part of their peptide sequence embedded in the hydrophobic region of the membrane.
GO:0016787	Hydrolase activity	Catalysis of the hydrolysis of various bonds, e.g. C-O, C-N, C-C, phosphoric anhydride bonds, etc. Hydrolase is the systematic name for any enzyme of EC class 3.
GO:0005634	Nucleus	A membrane-bounded organelle of eukaryotic cells in which chromosomes are housed and replicated. In most cells, the nucleus contains all of the cell's chromosomes except the organellar chromosomes, and is the site of RNA synthesis and processing. In some species, or in specialized cell types, RNA metabolism or DNA replication may be absent.
GO:0008270	Zinc ion binding	Interacting selectively and non-covalently with zinc (Zn) ions.
GO:0005524	ATP binding	Interacting selectively and non-covalently with ATP, adenosine 5'-triphosphate, a universally important coenzyme and enzyme regulator.
GO:0000166	Nucleotide binding	Interacting selectively and non-covalently with a nucleotide, any compound consisting of a nucleoside that is esterified with (ortho)phosphate or an oligophosphate at any hydroxyl group on the ribose or deoxyribose.
GO:0005515	Protein binding	Interacting selectively and non-covalently with any protein or protein complex (a complex of two or more proteins that may include other nonprotein molecules).
GO:0016020	Membrane	A lipid bilayer along with all the proteins and protein complexes embedded in it an attached to it.
GO:0046872	Metal ion binding	Interacting selectively and non-covalently with any metal ion.
