# Supplementary material for: B chromosomes of multiple species have intense evolutionary dynamics and accumulated genes related to important biological processes
Source: BMC Genomics. 2020 Sep 23;21:656. doi: 10.1186/s12864-020-07072-1 (PMC7509943; doi:10.1186/s12864-020-07072-1)
Supplement: Supplementary file 3 — Additional file 3: Figure S1. A workflow of steps applied in the present study during the procedure of genomics analyses of B chromosomes in different species. Figure S2. Coverage plots of B-blocks of A. correntinus with remarkable difference in the reads coverage between 0B and 1B samples. Figure S3. Coverage plots of B-blocks of A. flavolineata with remarkable difference in the reads coverage between 0B and 2B samples. Figure S4. Identification of B chromosome genomic blocks (A) and their repeats contents (B) in A. flavolineata. Figure S5. Identification of protein-coding genes located in B chromosomes of the A. mexicanus, using the number of mapped reads that map to the CDSs found in the transcriptome, in the 0B (X axis) and 2B (Y axis). Each dot represents a coding sequence with only those labeled that recorded the log2 greater than 1.5. The plot is limited for 800 mapped reads to optimize the visualizations. Figure S6. Identification of protein-coding genes located in B chromosomes of the A. correntinus, using the number of mapped reads that map to the CDSs found in the transcriptome, in the 0B (X axis) and 1B (Y axis). Each dot represents a coding sequence with only those labeled that recorded the log2 greater than 1. The plot is limited for 800 mapped reads to optimize the visualizations. Figure S7. Coverage plots of (representative) coding sequences detected on the B chromosome of A. mexicanus using Log base 2 ratio. Each plot compares the reads depth of the transcript between 0B and 2B. Figure S8. Coverage plots of (representative) coding sequences detected on the B chromosome of A. correntinus using Log base 2 ratio. Each plot compares the reads depth of the transcript between 0B and 1B. Figure S9. Coverage plots of (representative) coding sequences detected on the B chromosome of A. flavolineata using Log base 2 ratio. Each plot compares the reads depth of the transcripts between 0B and 2B. Figure S10. Double FISH mapping of candidate blocks in A. flavoli [file 12864_2020_7072_MOESM3_ESM.pdf]

Supplementary Figures

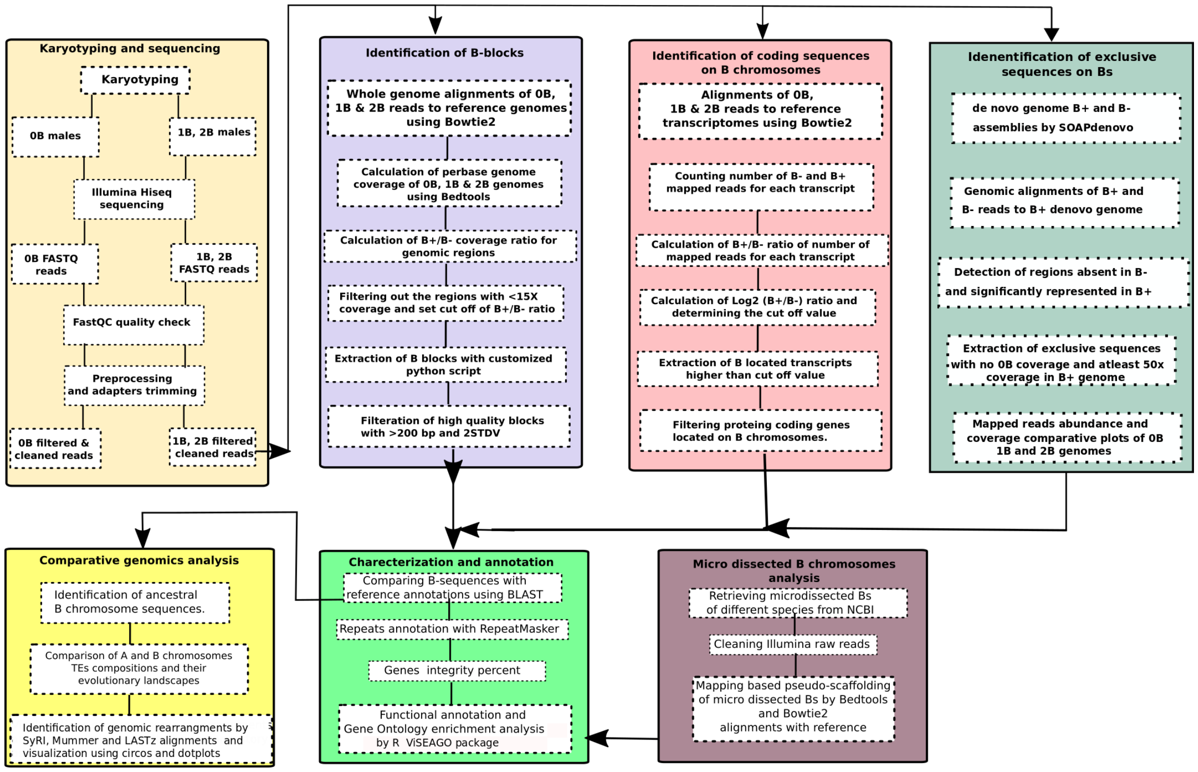

Fig. S1. A workflow of steps applied in the present study during the procedure of genomics analyses of B chromosomes in different species.

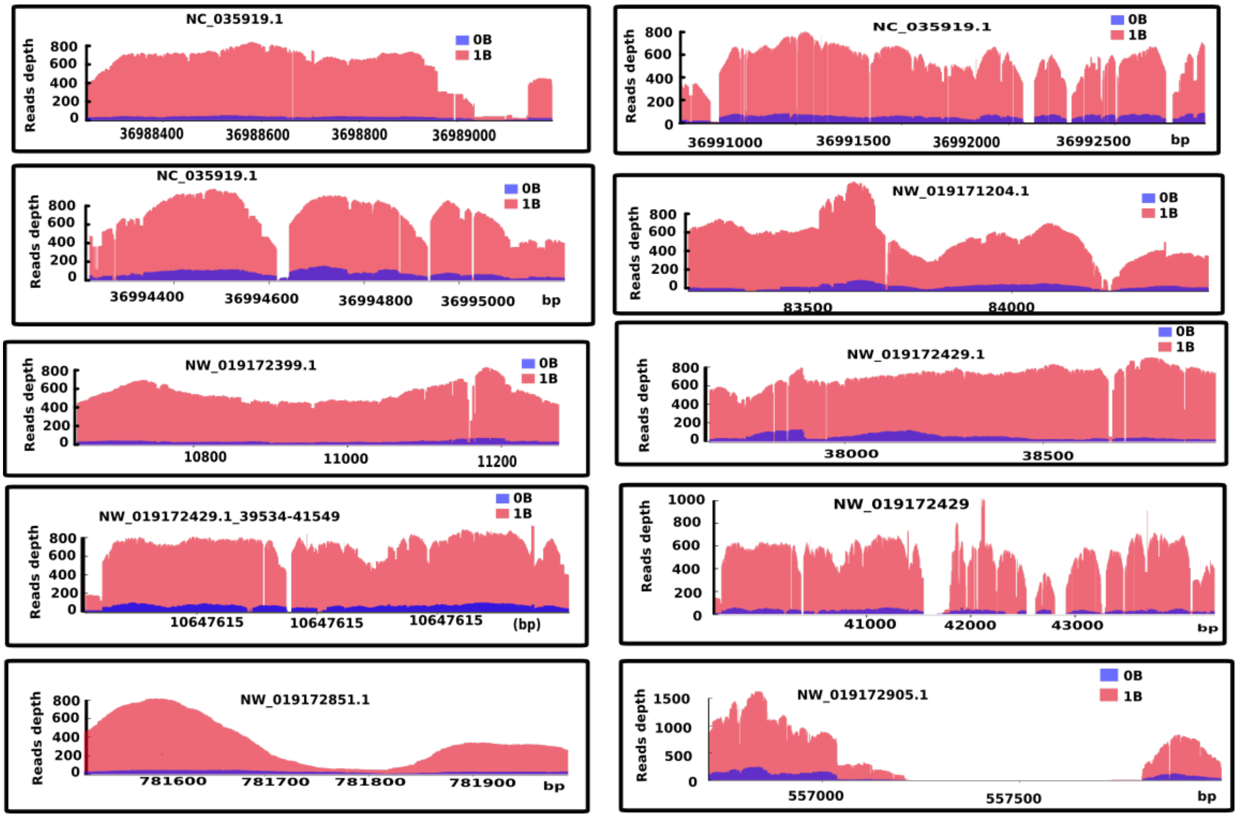

Fig. S2. Coverage plots of B-blocks of *A. correntinus* with remarkable difference in the reads coverage between 0B and 1B samples.

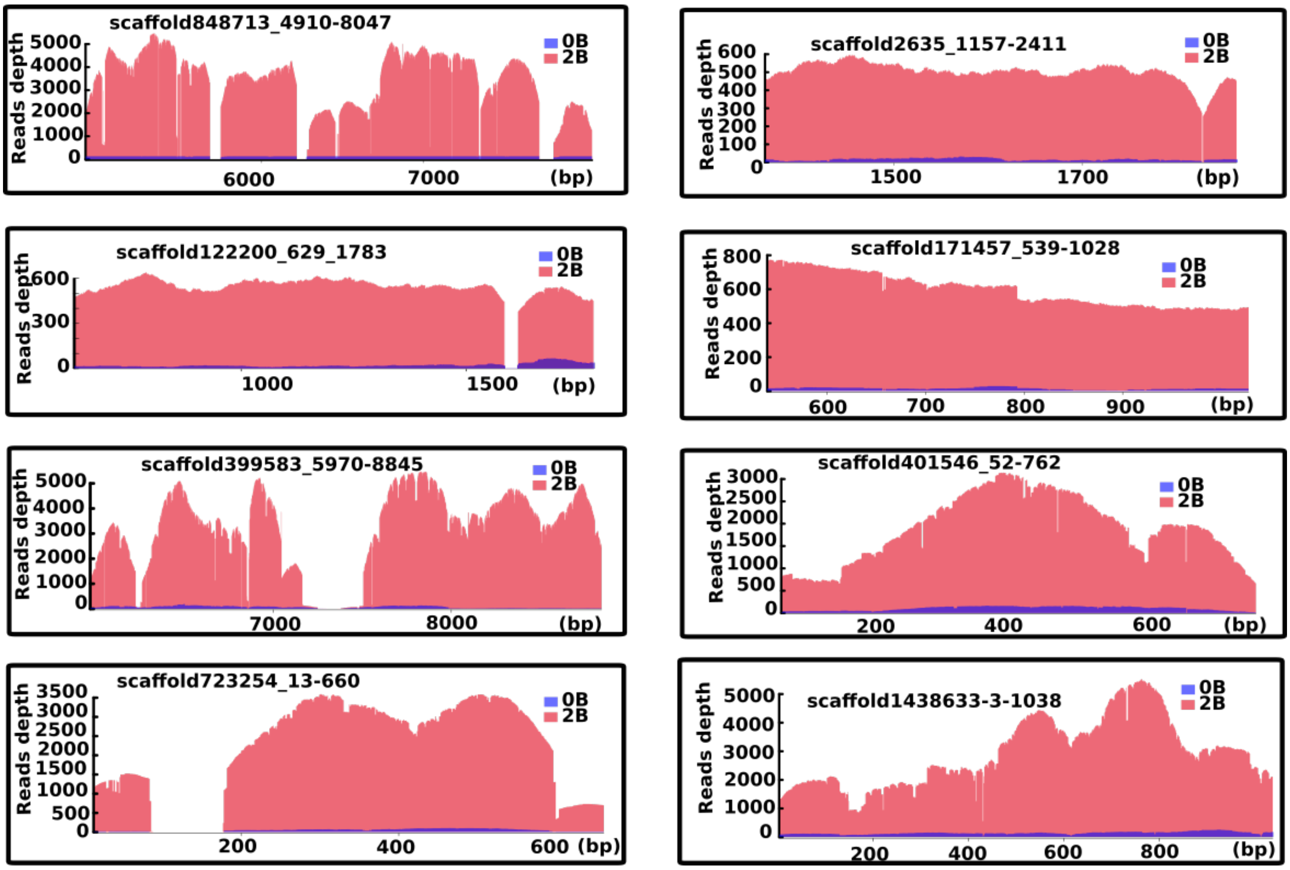

Fig. S3. Coverage plots of B-blocks of *A. flavolineata* with remarkable difference in the reads coverage between 0B and 2B samples.

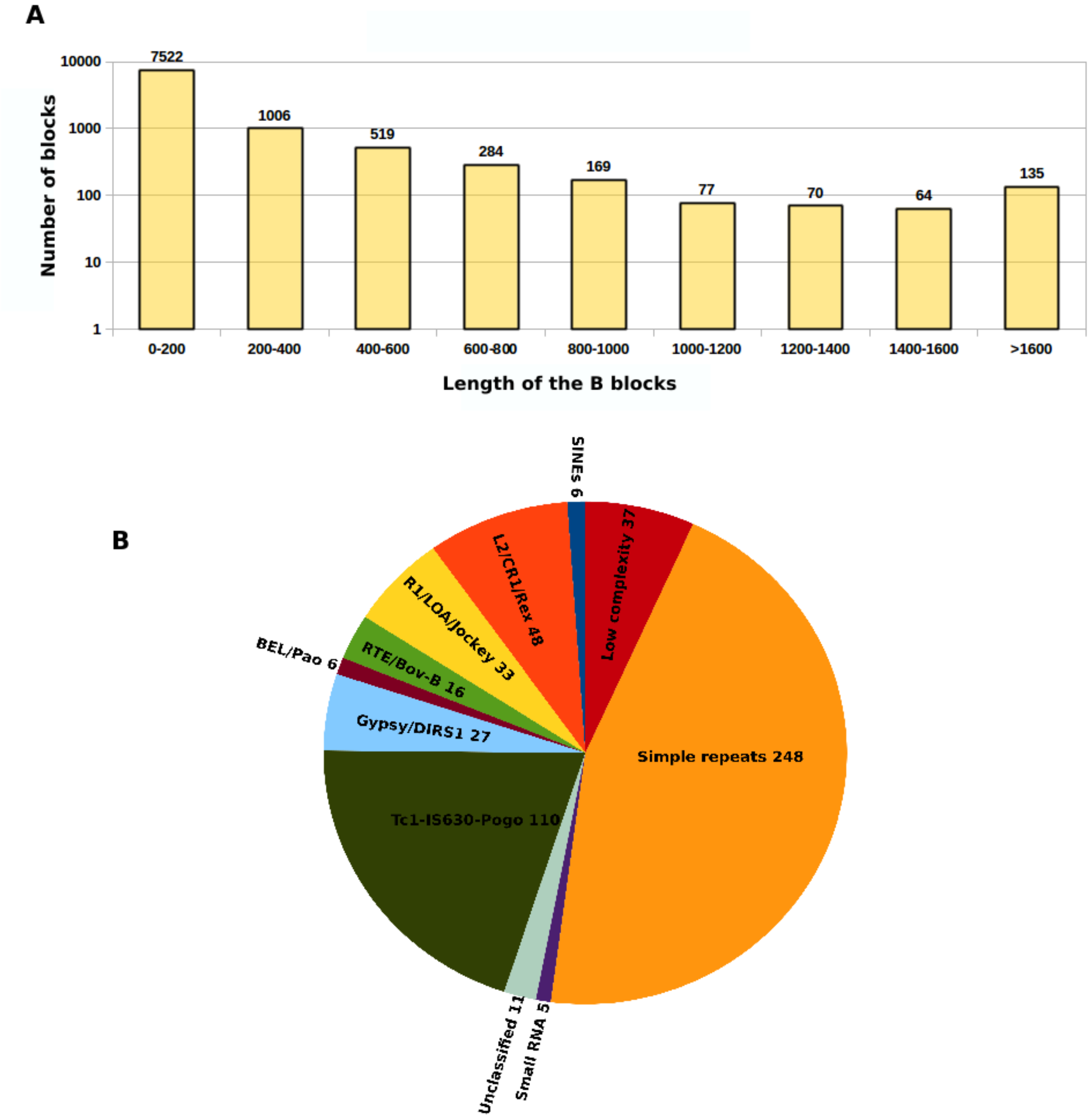

Fig. S4. Identification of B chromosome genomic blocks (A) and their repeats contents (B) in *A. flavolineata*.

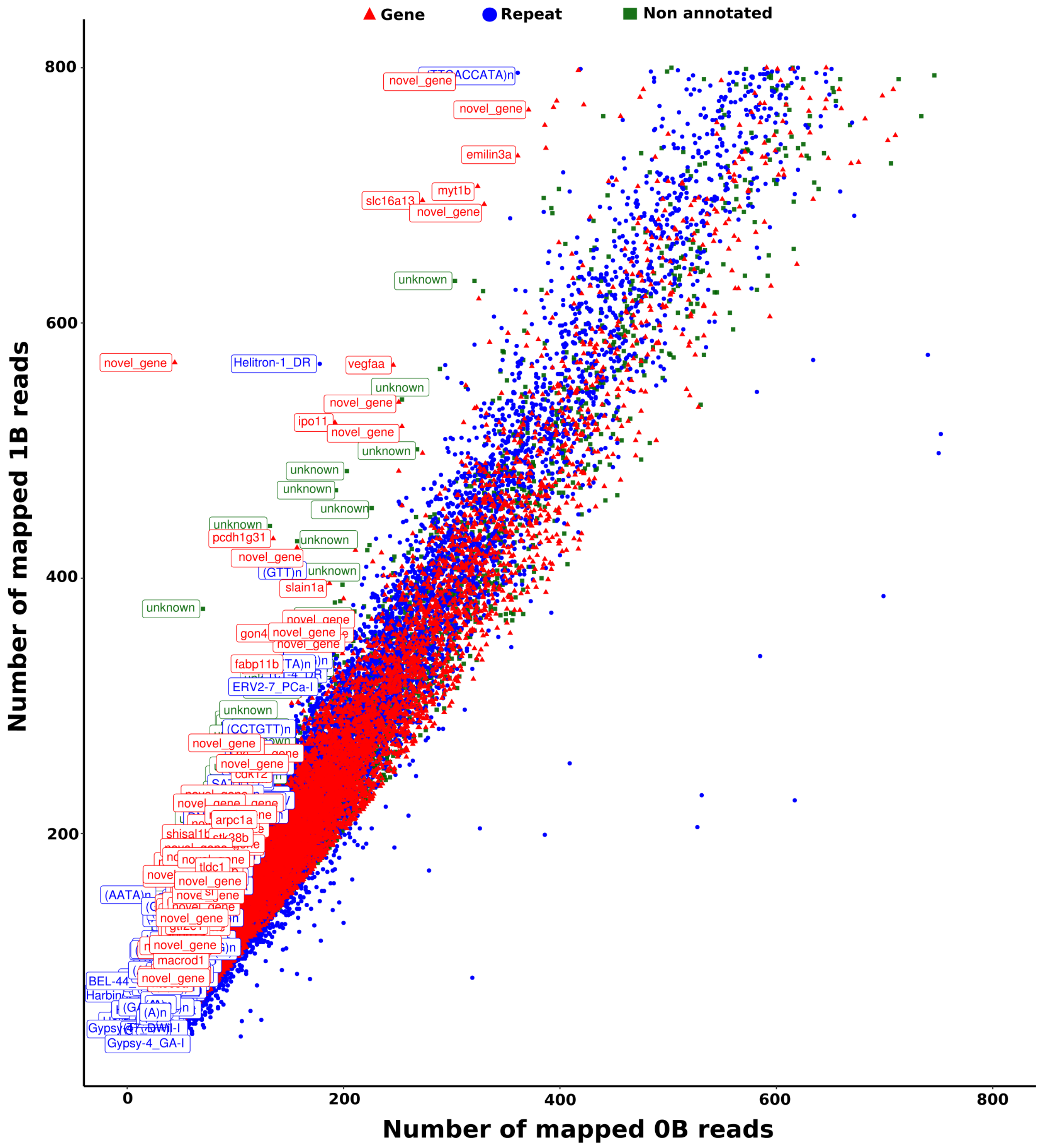

Fig. S5. Identification of protein-coding genes located in B chromosomes of the *A. mexicanus*, using the number of mapped reads that map to the CDSs found in the transcriptome, in the 0B (X axis) and 2B (Y axis). Each dot represents a coding sequence with only those labeled that recorded the log_2 greater than 1.5. The plot is limited for 800 mapped reads to optimize the visualizations.

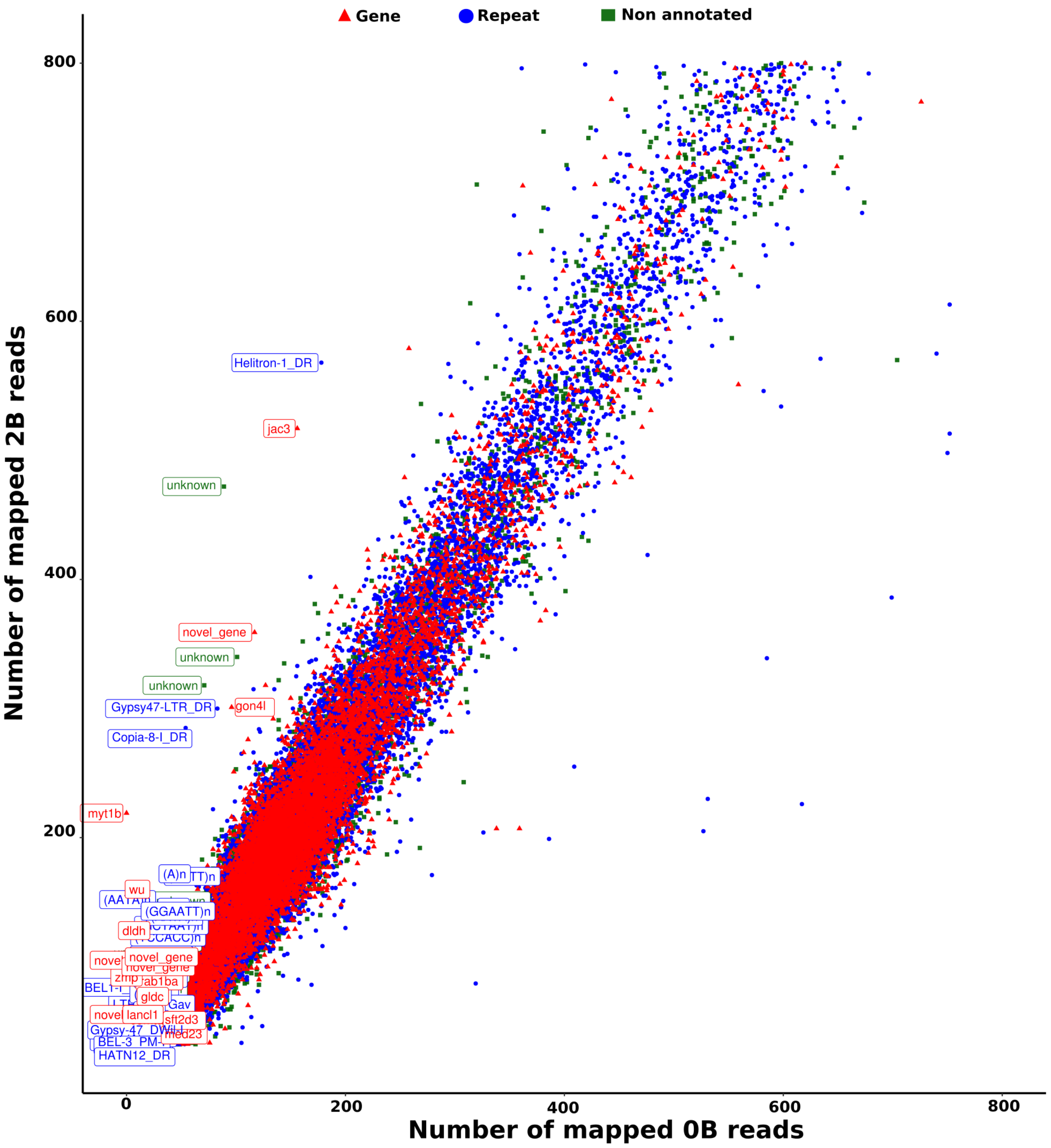

Fig. S6. Identification of protein-coding genes located in B chromosomes of the *A. correntinus*, using the number of mapped reads that map to the CDSs found in the transcriptome, in the 0B (X axis) and 1B (Y axis). Each dot represents a coding sequence with only those labeled that recorded the log2 greater than 1. The plot is limited for 800 mapped reads to optimize the visualizations.

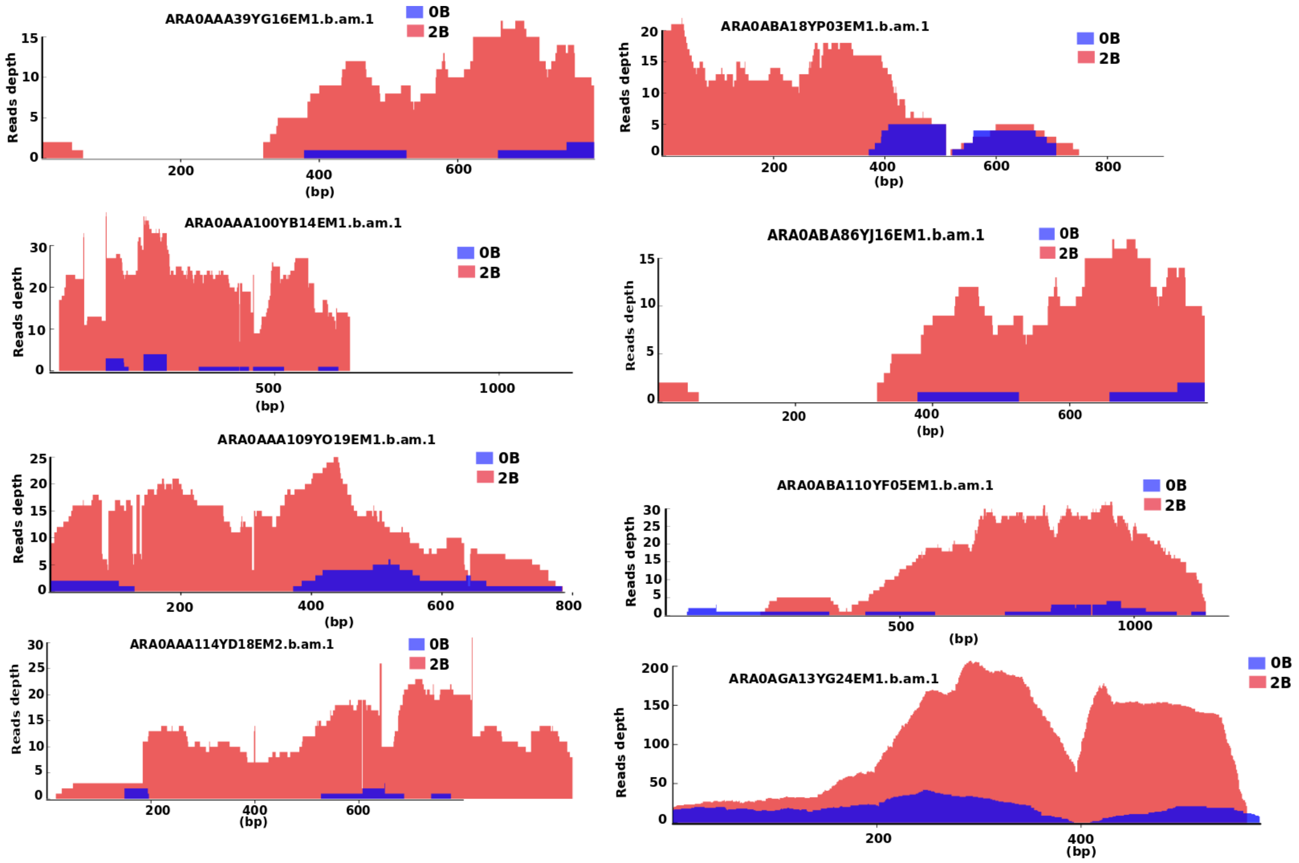

Fig. S7. Coverage plots of (representative) coding sequences detected on the B chromosome of *A. mexicanus* using Log base 2 ratio. Each plot compares the reads depth of the transcript between 0B and 2B.

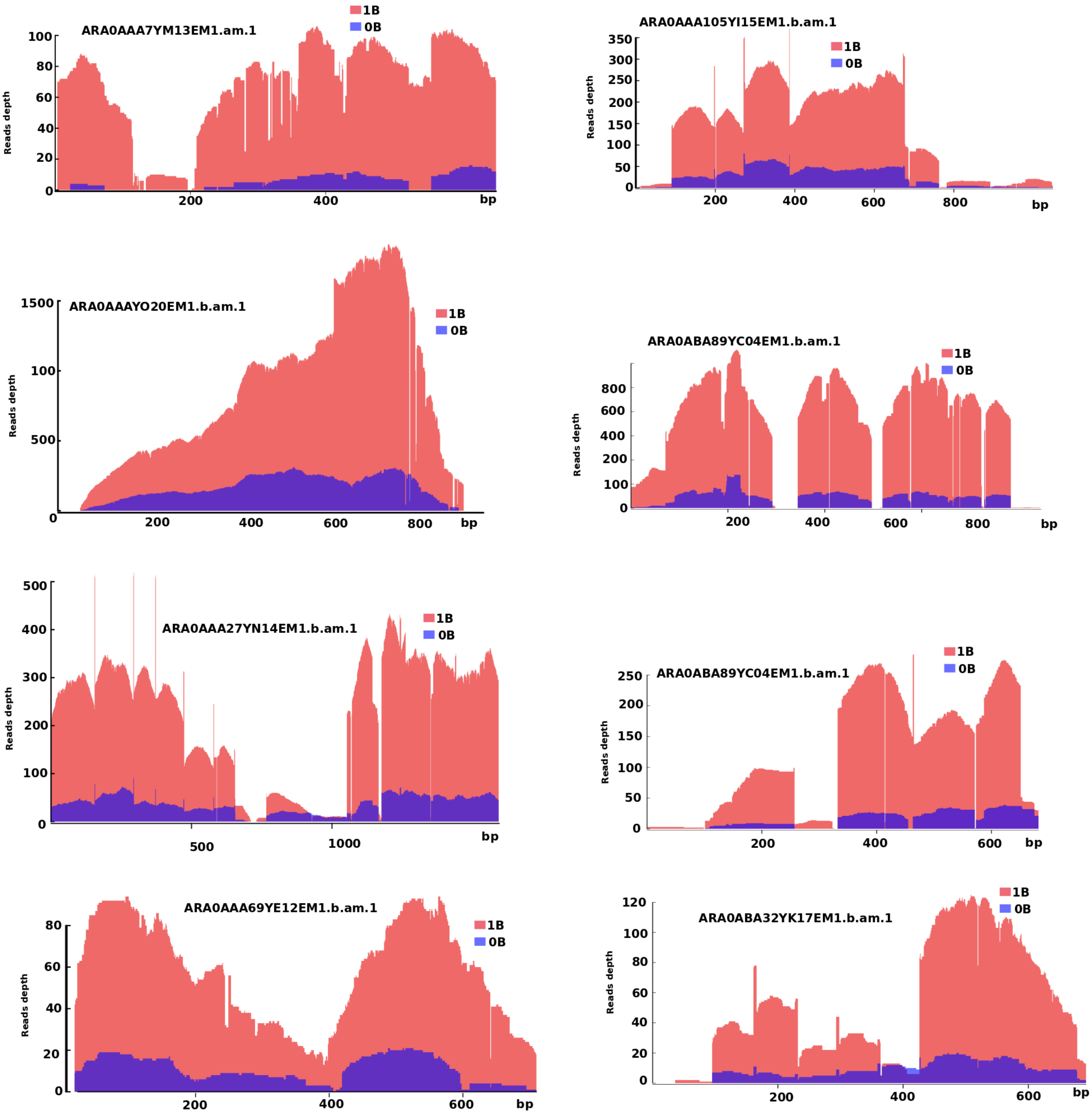

Fig. S8. Coverage plots of (representative) coding sequences detected on the B chromosome of *A. correntinus* using Log base 2 ratio. Each plot compares the reads depth of the transcript between 0B and 1B.

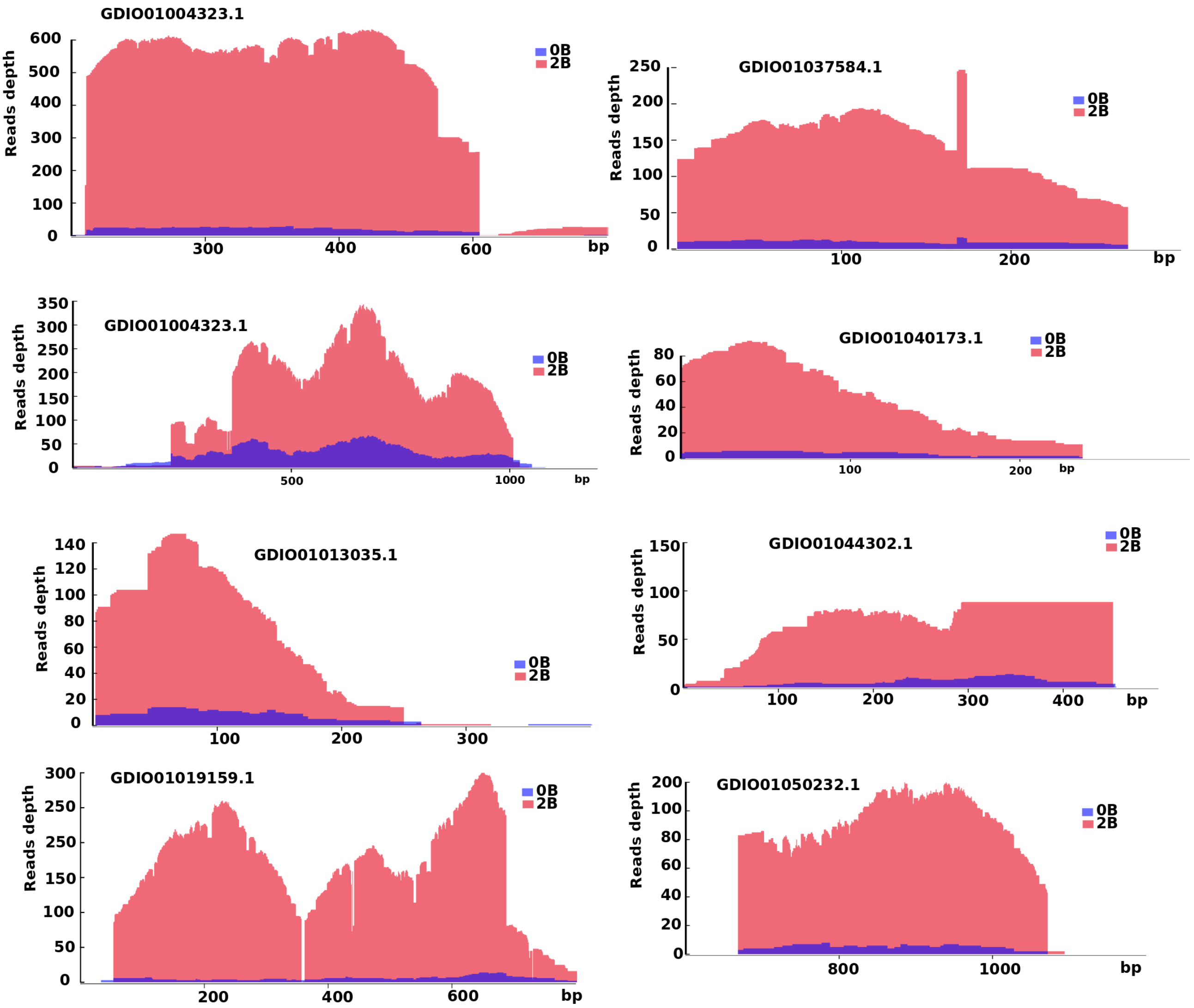

Fig. S9. Coverage plots of (representative) coding sequences detected on the B chromosome of *A. flavolineata* using Log base 2 ratio. Each plot compares the reads depth of the transcripts between 0B and 2B.

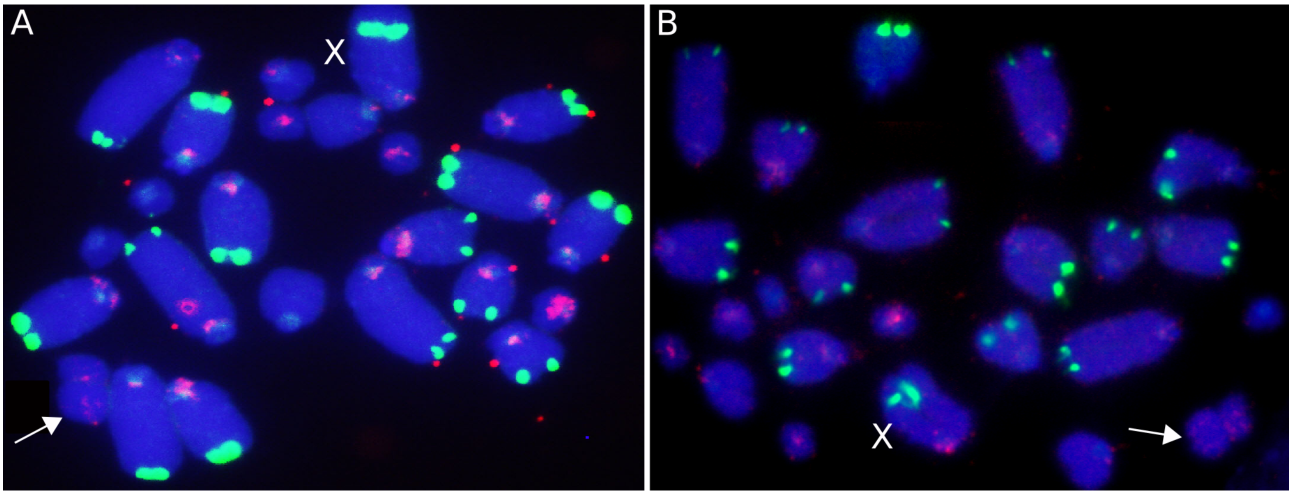

Fig. S10. Double FISH mapping of candidate blocks in *A. flavolineata*. Each panel represents mapping of two distinct blocks (7 and 14 – See Supplementary Table S2) labeled with digoxigenin (block 7 in red) and biotin (block 14 in green). A scattered pattern of markings of block 7 can be observed for the B chromosomes (white arrows), whereas telomeric and centromeric associated signals of blocks 7 and 14 are obvious for different A and X chromosomes.

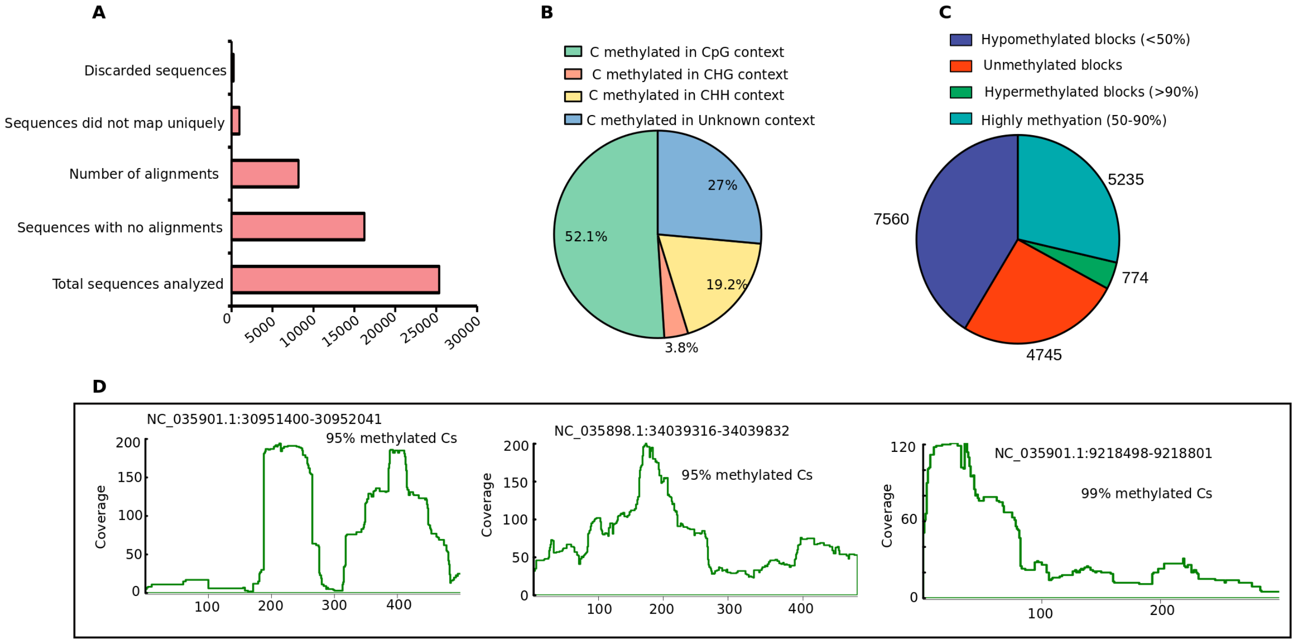

Fig. S11. The methylation profile of microB blocks in *A. mexicanus. (***A**) Graph shows the number of Bisulphite reads analyzed with B blocks and alignments. (**B**) Pie chart represents percentage of B block methylated Cs in different contexts with the highest percent in CpGs regions. (**C**) Pie chart highlights the number of hypomethylated (less than 50% methylated Cs), unmethylated, highly methylated and hypermethylated (more than 90% methylated Cs) B chromosome blocks. (**D**) Bisuphite coverage plots of hypermethylated blocks are shown as examples. Refer to Supplementary datasets (excel) for a complete list of methylated blocks with methylation level.

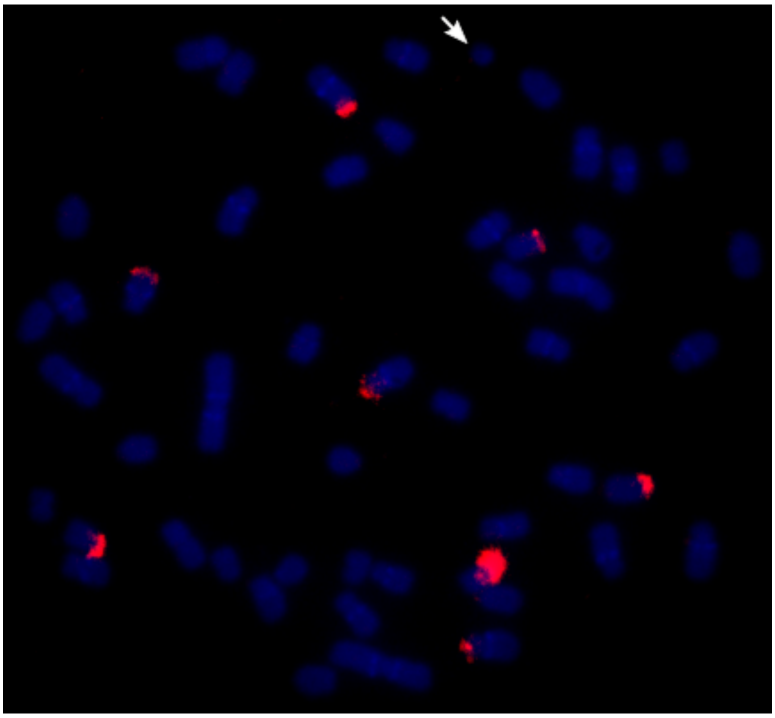

Fig. S12. FISH mapping of 45S rDNA (red labeling) in *A. mexicanus.* The micro B is indicated with an arrow showing no sign of 45S rDNA.

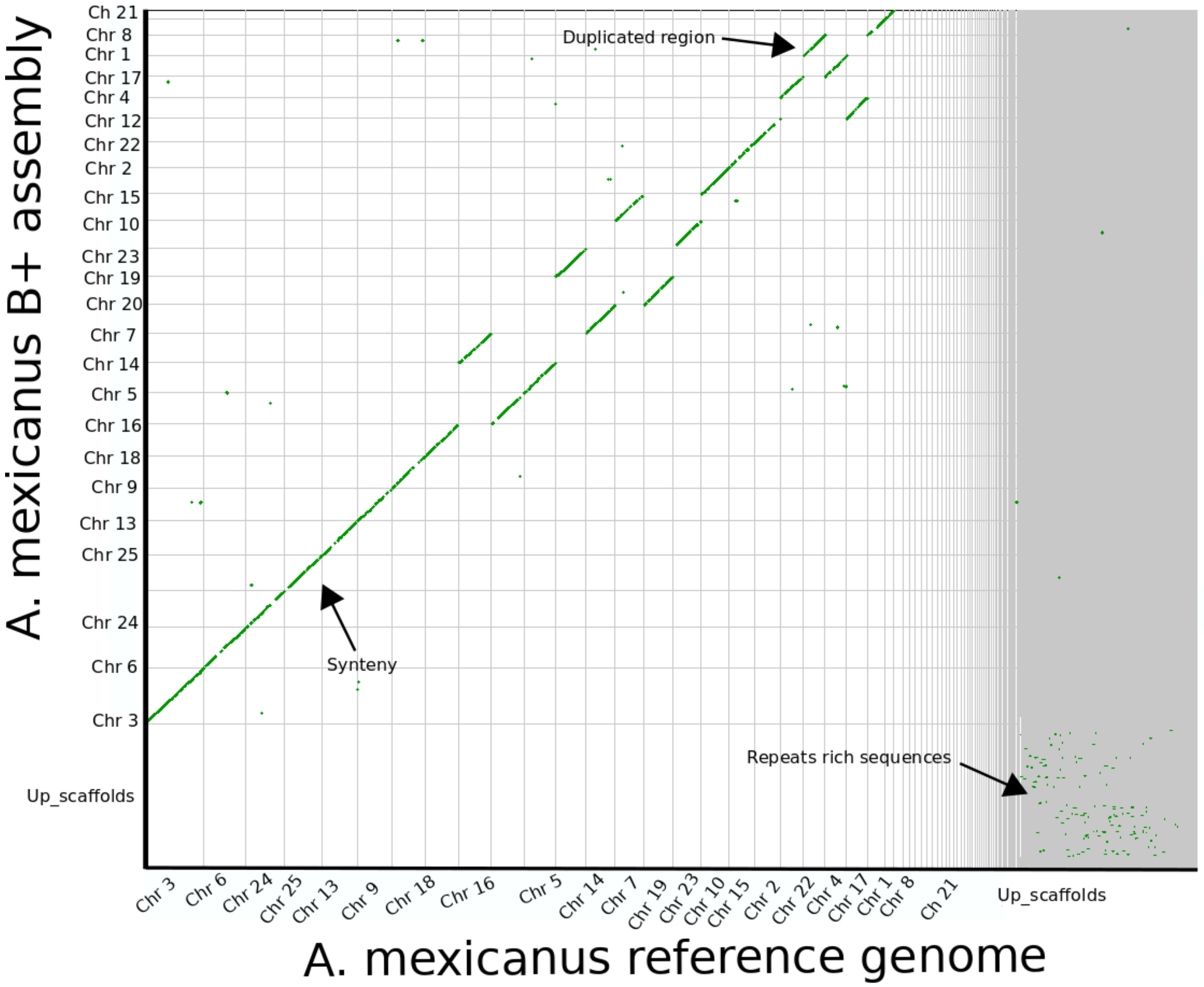

Fig. S13. Synteny dotplot of whole genome aligments between de novo B+ assembly and reference masked (coding) genome of *A. mexicanus*.

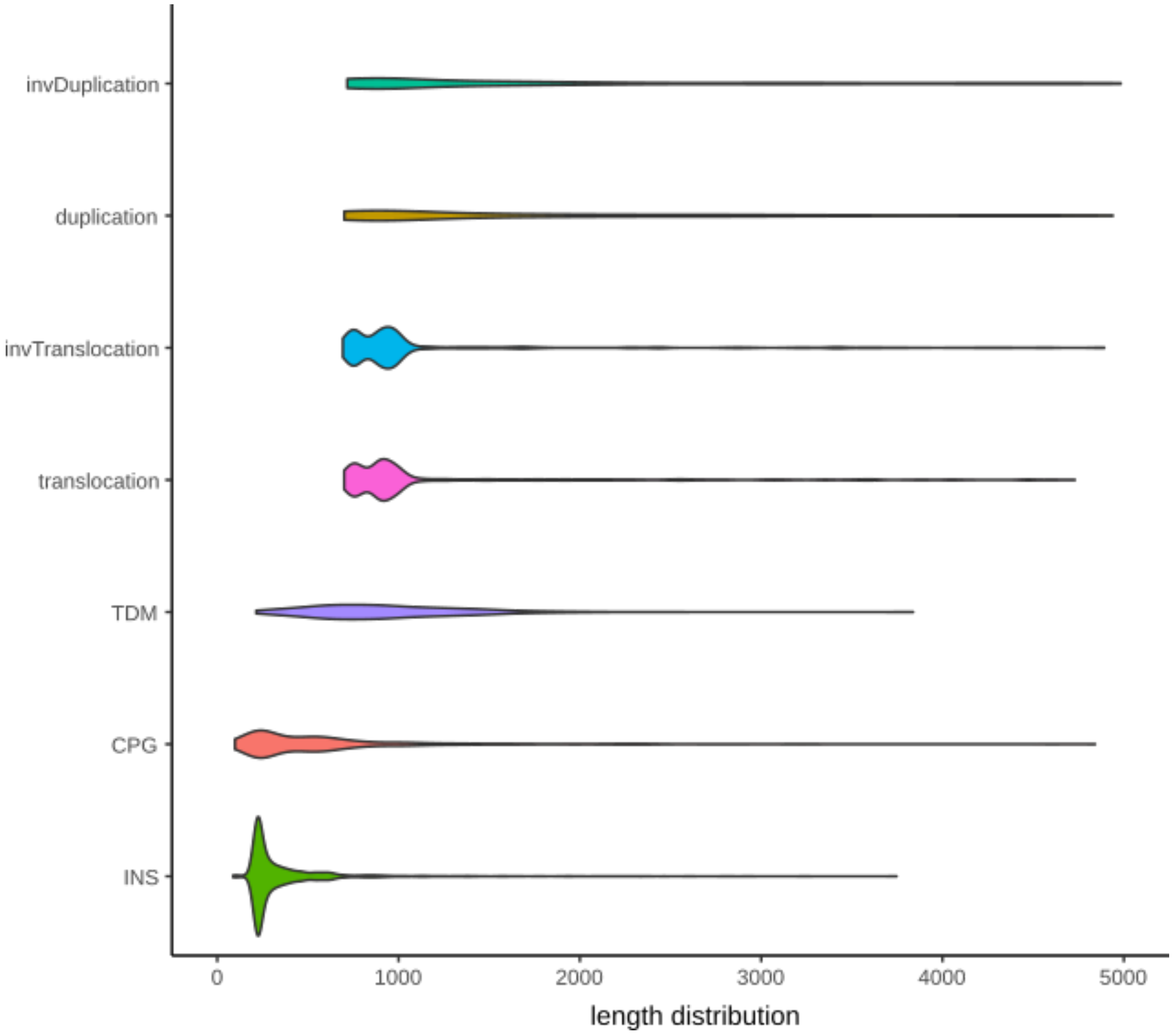

Fig. S14. Violin plots show the distribution of length for various types of genomic rearrangements from 0-5000 bp range.

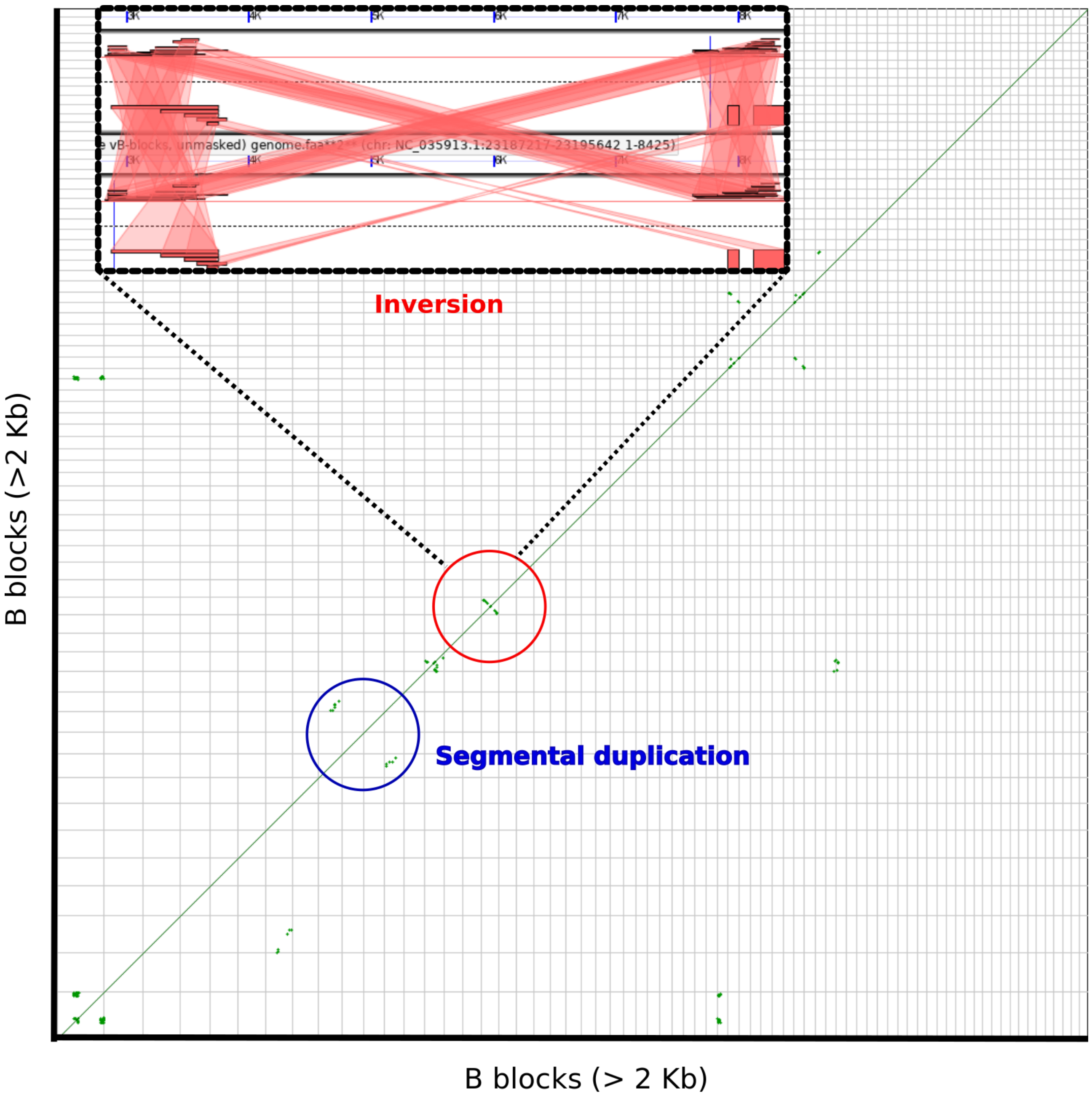

Fig. S15. Synteny dotplot (synmap version, unfiltered) of self alignments of B blocks (*A. mexicanus*).

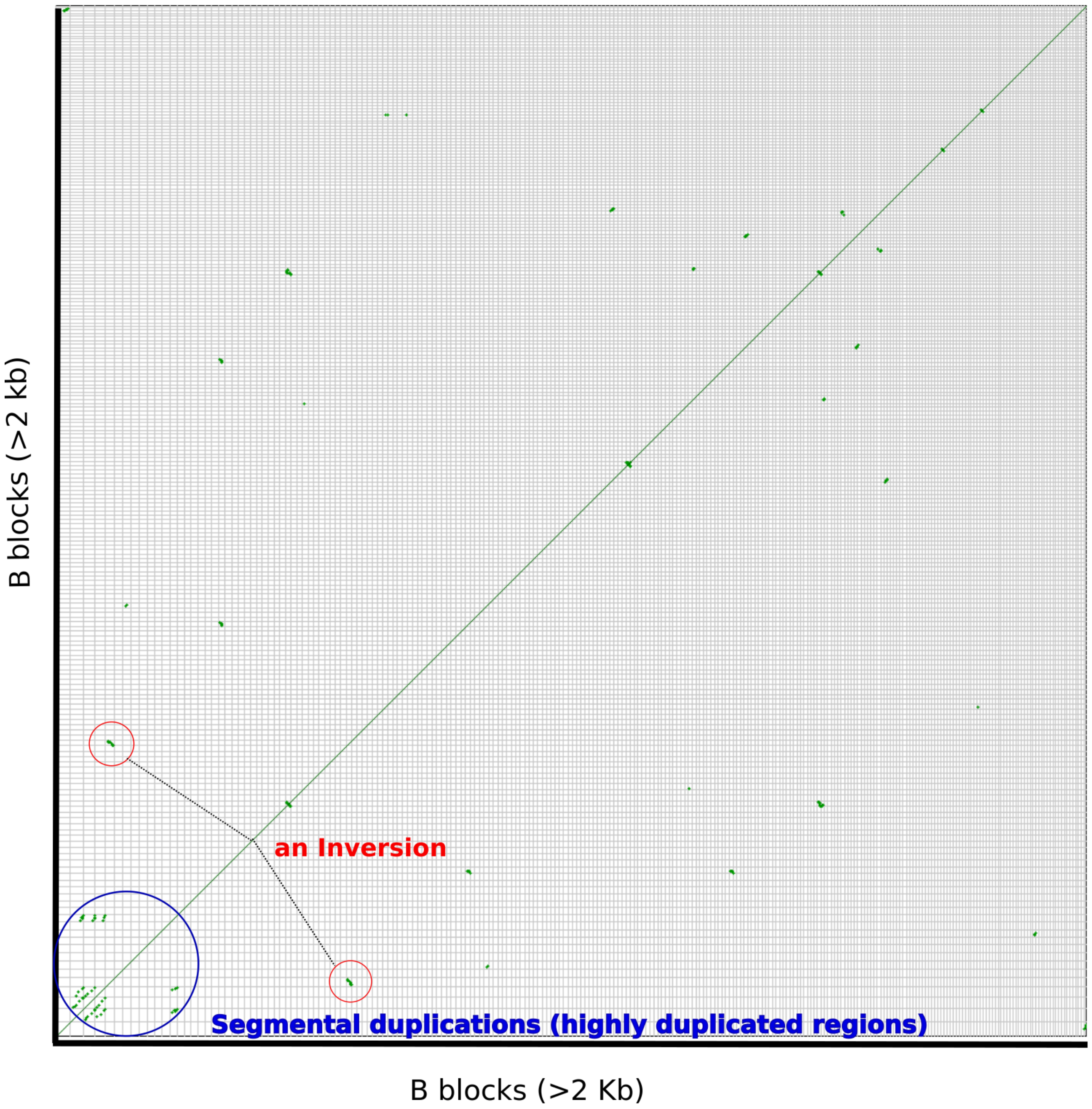

Fig. S16. Synteny dotplot (synmap version, unfiltered) of self alignments of B blocks (*A. correntinus*).

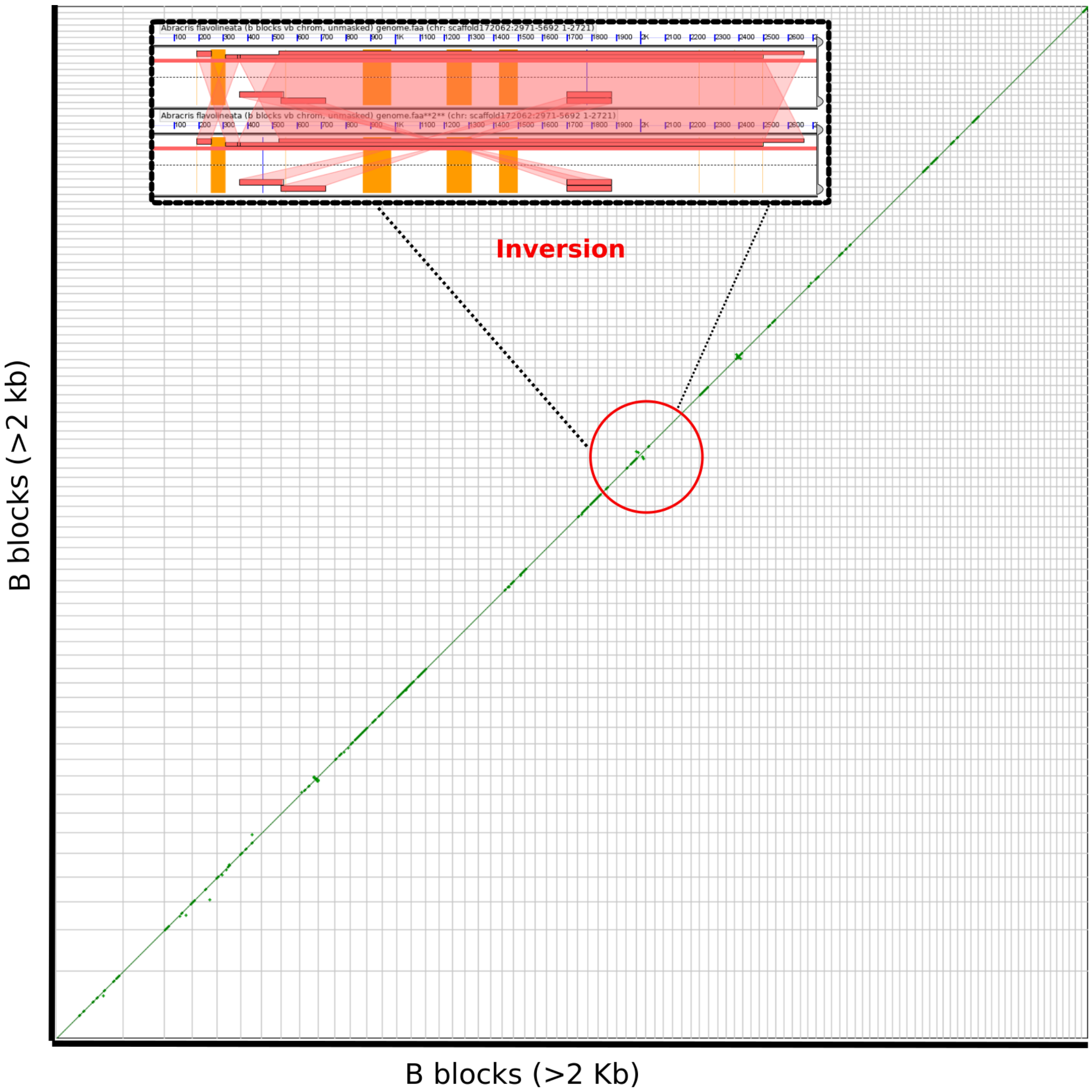

Fig. S17. Synteny dotplot (synmap version, unfiltered) of self alignments of B blocks (*A. flavolineata*).
